# Conflict management as a nurse’s competency in the surgical ward

**DOI:** 10.1590/1980-220X-REEUSP-2025-0426en

**Published:** 2026-03-06

**Authors:** Ana Laura Pircio, Laura Andrian Leal, Divanice Contim, Silvia Helena Henriques, Iasmin Gabrielli da Silva, Ivaneia Alves Pereira Sobrinho, Luan Gagossian Savóia, Silvio Luiz de Flório, Josué Souza Gleriano, Andrea Bernardes, Bethania Ferreira Goulart, Priscila Linardi Guimarães

**Affiliations:** 1 Universidade Federal do Triângulo Mineiro, Departamento de Enfermagem, Uberaba, MG, Brazil.; 2 Universidade de São Paulo, Escola de Enfermagem de Ribeirão Preto, Ribeirão Preto, SP, Brazil.; 3 Centro Universitário Barão de Mauá, Departamento de Psicologia, Ribeirão Preto, SP, Brazil.; 4 Universidade do Estado de Mato Grosso, Departamento de Enfermagem, Tangará da Serra, MT, Brazil.; 5 Universidade Federal do Triângulo Mineiro, Departamento de Enfermagem em Educação e Saúde Comunitária, Uberaba, MG, Brazil.; 6 Prefeitura Municipal de Sertãozinho, Sertãozinho, SP, Brazil.

**Keywords:** Dissent and Disputes, Negotiating, Nursing, Professional Competence, Perioperative Nursing

## Abstract

**Objective:**

To analyze the perception of nurses in a surgical ward regarding conflict management as a professional competence and the strategies for managing it.

**Method:**

This is an exploratory and qualitative study. The setting was a public hospital in Minas Gerais. The study population consisted of nurses from the surgical ward, with data collected between June and December 2024. Inductive content analysis was used. The study was approved by the institution's ethics committee.

**Results:**

Categories emerged: “Factors associated with conflict management competence”, related to interprofessional, ethical, and communication skills; “Causes of conflicts”, linked to work overload and other factors; and “Management strategies”, such as team meetings and respectful dialogue, which contribute to improving the quality of care.

**Conclusion:**

Nurses recognize conflict management as an essential competency in their professional practice, combining technical, emotional, and communication skills. The management strategies identified reveal efforts to promote more harmonious work environments and improve the quality of care provided.

## INTRODUCTION

The unique nature of hospital organizations has been highlighted by the provision of care to patients in increasingly critical conditions. This demands specific skills from healthcare professionals to cope with constant technological innovations, varied working conditions, behavioral changes, and the growing demands of patients, sometimes leading to transformations in their work processes^(1)^.

Among these skills, the ability to identify and address conflicts stands out, since such situations impact the organizational well-being of the healthcare team, as they are intrinsically related to the coexistence of individuals who have different values, beliefs, backgrounds, and opinions^(2)^.

Conflict is defined as a process in which an individual, consciously and intentionally, makes efforts to impede the actions of another, establishing some kind of obstacle that compromises the achievement of their goals and interests. Disagreements, disputes, and conflicts within healthcare teams are frequent and can result in reduced professional productivity, negatively impacting the care and treatment provided to patients. Therefore, the acquisition of skills by professionals becomes essential^(3)^.

It is known that nurses, in the hospital setting, perform multiple duties that are linked both to direct patient care and to managerial functions^(4)^. From this perspective, competencies are understood as an integrated set of knowledge, skills, attitudes, and values. Specifically in the case of nurses, such competencies have been related to the ability to combine information, conduct, and principles, as well as technical skills, clinical reasoning, conflict negotiation, and effective communication^(5)^.

Thus, hospital nurses need to deal with multiple demands and tasks characterized by a high degree of requirement and responsibility, which, depending on their experience, scientific knowledge, and professional attitude, may or may not favor the emergence of conflictual relationships in this work context.

Therefore, it is essential that nurses develop skills focused on conflict management, to ensure the quality of care provided and the maintenance of organizational health. In the development of the work process, it is essential that the nurse demonstrates proficiency in communication, observation, active listening, critical thinking, and empathy, to understand all facets of a dissenting opinion^(6)^.

Consequently, it is observed that, in complex sectors such as surgical wards, this issue constitutes a substantial problem that directly impacts the quality of care, due to the inherent complexity of the environment, characterized by high patient turnover, intensive care demands, and the need for continuous coordination between different specialties.

In this sphere, direct and uninterrupted care is provided by the nursing team, which demands from nurses not only technical skills, but also managerial and relational skills to deal with adverse situations, often intensified by pressure for immediate results, scarcity of human resources, and work overload^(7)^.

The surgical ward plays a predominantly assisting and operational role, focused on the clinical monitoring of patients in the pre- and post-operative periods, and is generally organized according to the specialty or type of surgical procedure. Because it is a more restricted area^(7)^, a lack of scientific evidence to problematize its dynamics and provide strategies for its proper management^(4,5)^ is observed.

Accordingly, investigating the perceptions of nurses working specifically in the surgical ward allows for a deeper understanding of the challenges and strategies employed in addressing conflicts in this context, contributing to the improvement of professional training and the development of institutional policies aimed at promoting more collaborative and healthy work environments, including other sectors.

For that reason, this study presents the following questions: Does the nurse perceive conflict negotiation as a professional skill? How does conflict negotiation by nurses occur in the context of the surgical ward? Are there strategies for managing this?

By focusing on a hospital sector, such as the surgical ward, the study examines an environment where conflicts are not only frequent, but potentially more intense, given the diversity of professionals involved, the pressure for immediate results, and the care of patients in critical situations^(7)^. This choice allows us to grasp nuances that might go unnoticed in less dynamic contexts, revealing how nurses deal with tensions that directly affect the quality of care, patient safety, and organizational climate.

From this perspective, this investigation will provide imminent benefits to the quality of nursing care, given the possibilities of reflecting on the topic and listing propositons that result in a positive organizational atmosphere.

Furthermore, its justification lies in the fact that it is a pressing issue that demands further study by professionals in the field, since the hospital context reveals complex situations in which the ability to negotiate conflicts can result in positive outcomes for both the care provided to the user and the institution.

Thus, the objective of this study was to analyze the perception of nurses in a surgical ward regarding conflict management as a professional competence and the strategies for handling it.

## METHOD

### Design of Study

This is an exploratory study with a qualitative approach to data analysis. Qualitative research makes it possible to investigate perceptions, behaviors, cultures, and social relationships, allowing for a holistic and detailed understanding of the realities experienced by the participants^(8)^. The article was prepared in accordance with the *Consolidated Criteria for Reporting Qualitative Research* (COREQ)^(9)^ guidelines.

### Local

The study setting was a public hospital in the state of Minas Gerais. The selected hospital is a reference within the Brazilian Public Health System (*SUS*) at the Municipal, State, and National levels, encompassing care for patients requiring hightech treatments. Specifically, the surgical ward was selected, which occurred randomly.

### Population and Selection Criteria

The study population consisted of nurses from the surgical ward of the selected hospital. The reason for selecting the population from this sector is that it encompasses a variety of professional categories working together for the benefit of the patient. In this context, nurses assume a central role in coordinating care, supervising the technical team, and mediating interpersonal relationships, which makes them particularly exposed to conflict situations.

All nurses working in the surgical ward who had at least six months of experience were included and invited on a voluntary basis, considering that these professionals may have dealt with and managed conflict issues in the workplace. Professionals with less than six months of experience and/or those who were only providing occasional coverage for the sector were not included, as they were not permanent members of the surgical ward team. It should be noted that the department had 17 nurses.

### Data Collection

It is crucial to highlight that the nurses were personally invited through a letter of invitation from the study’s first author, who did not exert any power or influence over the recruited participants. The benefits of the research were explained to the participants, who were also informed that participation was voluntary.

For data collection, individual semi-structured interviews were conducted, with the interview guide consisting of two parts: The first contains the nurses’ sociodemographic data, and the second contains the following questions: Is conflict negotiation a professional skill for nurses? Discuss and give examples of situations; How do you conduct conflict negotiation in the context of the surgical ward? Reflect: Were you prepared to deal with these issues?

The interviews lasted an average of 15 minutes and were conducted by the first author, who had been previously trained by her supervisor; they took place individually during the nurses’ break time at work, according to the participants’ availability. Data collection took place from June to December 2024.

Before the interview began, an explanation was given about the research topic, its objectives, and the relevance of participating in the study, as well as a guarantee of anonymity, seeking to alleviate concerns about any future exposure. Furthermore, the interviews were audio-recorded and subsequently transcribed manually, without the aid of software.

### Data Analysis and Treatment

The corpus of the evaluation consisted of transcribed material from the recordings, and the inductive content analysis method was used, based on the thematic analysis modality, supported by the theoretical framework^(2,3)^.

Thus, the phases of data interpretation and description in this study were considered, such as: becoming familiar with the data, generating initial codes, searching for themes, reviewing themes, defining and naming themes, as well as producing the analysis report^(10)^. Therefore, an analysis of the speeches was carried out using the recordings, and the data were organized into categories to better discuss the subject.

It is important to emphasize that the participants were identified by the letter E for nurse in Portuguese (*Enfermeiro*), and received a sequential Arabic numeral, thus guaranteeing the anonymity of their statements. Thus, they were referenced as E1, E2, and so on. No Artificial Intelligence was used.

### Ethical Aspects

The study was submitted to and approved by the Research Ethics Committee of the selected Hospital of the Universidade Federal do Triângulo Mineiro (HC-UFTM), under opinion number 6,931,316 of 2024.

## RESULTS

Thirteen nurses participated in the study, predominantly female, 12 (92.3%), with ages ranging from 27 to 45 years, with an average age of 36.8 years. Regarding origin, the majority, seven (53.8%), were from the state of Minas Gerais, followed by two (15.3%) from Bahia, two (15.3%) from São Paulo, one (7.7%) from Pernambuco, and one (7.7%) from Maranhão. The participants’ length of service in the surgical ward ranged from six months to nine years, with an average of 2.7 years.

The time since graduation ranged from six months to 20 years, with an average of 11.7 years. Regarding work in other hospital sectors, seven (53.8%) reported not having worked in other departments, and six (46.1%) reported having worked in other sectors, such as neurology, orthopedics, pediatrics, adult and pediatric emergency room, and coronary, adult, and neonatal Intensive Care Unit (ICU).

It should be noted that, of all participating nurses, 11 (84.61%) held a graduate certificate in various areas, such as Surgical Nursing, Urgency and Emergency, Cardiology, Intensive Care, and others; moreover, it should be underscored that many professionals had more than one specialization.

Based on the interview data, categories were established for organizing the results, as follows: “Factors Associated with Conflict Management Competence”; “Causes of Conflicts”; and “Strategies for Conflict Management: Individual and Organizational”.

### Factors Associated with Conflict Management Competence

In this category, it is important to contextualize that the participating nurses stated that they deal daily with a multitude of demands and interpersonal relationships related to the subordinate technical team, patients with their companions, and all other professionals who make up the multidisciplinary team, since all health care that takes place in the sector falls on their shoulders, thus they are entrusted with the function of leading and managing all situations.

As a consequence, the nurses described conflict management competence as the ability to mediate, manage, deal with, mitigate, and resolve adverse situations that arise during their shift. Other associated factors mentioned were the need to build interpersonal and interprofessional relationships with impartiality and assertive communication, as evidenced in the accounts below:

*Conflict management, I think, is how you deal with disputes that arise in your work; it’s about trying to smooth things over, whether with your own peers, with nursing technicians, or with other professionals.* (E6)

*It’s about knowing how to handle daily situations, generally between patients, with professionals, and among professionals themselves. We need to be flexible, have good interpersonal relationships, as well as relationships with the team, patients, caregivers, and all hospital professionals, and above all, have clear communication and be impartial. We can’t have a side or preference; we need to know how to analyze the situation.* (E7)

The ability to identify and conceptualize conflict, as well as understand that its management is an essential skill for hospital nurses, proves indispensable, since it prevents the deterioration of personal relationships and reduces the need to access higher levels of hospital management.

*There’s no way to prevent it from happening, but in my opinion, managing it means at least achieving... How can I put it? Don’t let things get worse, don’t let this conflict escalate, take it forward, to a long-term. Try to resolve it there in the most timely way possible.* (E2)

*You need to manage it so that it doesn’t become something bigger, so that it doesn’t achieve other proportions. I think that’s what conflict management is all about: trying to resolve things more quickly, so you don’t end up creating other conflicts or other situations on top of one conflict. I think that would be it.* (E1)

### Causes of Conflicts

The most common causes related to the emergence of dissension/conflicts in the surgical ward were cited, the main one being work overload due to absenteeism and sick leave. However, the night shift team reports having an additional aggravating factor that contributes to this overload, since during their work period they do not have enough academics, residents, doctors, or even the Technical Manager (RT) of the sector.

Furthermore, other causes of conflict described were related to the technical team having a perspective of injustice regarding the delegated functions and a disharmonious interpersonal relationship. Furthermore, participants also reported that when team members have personal problems or dual employment, they arrive at work tired and stressed, which often results in transferring this tension to colleagues or patients. Thus, these professionals begin to act disrespectfully, rudely, and explosively, and sometimes ignore the demands of the caregivers, which contributes to increased friction and disagreements.

Moreover, uncooperative companions were also cited as potential sources of conflict. Finally, another issue related to the multidisciplinary team is the lack of interprofessional care, since fragmented and uncooperative care leads to communication failures, especially between doctors and nurses, as explicitly stated in the testimonies:

*Something that involves a lot of conflict management is the division, the nursing division. It’s one of the things I’ve noticed most recently, due to a lack of professionals. So, often we need to have an organization that ends up not being the way it would be in the legislation, this division, because we don’t have enough professionals.* (E4)

*I think there’s more conflict at night than during the day. During the day there are more people to help, there are more professionals. The doctor arrives, and he explains everything. The academic arrives and says it all. The resident arrives and tells all. The psychologist arrives, then... So, there are so many professionals that it ends up not overloading the team with complaints as much. Now at night, only we can listen. The more you go there, the more they complain. There’s no way to call the doctor or resident for those situations. That’s when the team gets more tired and overworked.* (E3)

*Sometimes the technician, who works in multiple places, arrives already stressed, and also dealing with a family problem, and ends up taking it out on the patient.* (E11)

*There are 7 nursing technicians for 44 or 45 patients, that’s very few. There are far too few staff for too many patients. So there’s no way around it. Even if I wanted to, I couldn’t even put a technician in every ward.* (E9)

*Because there’s so much workload, I see that everyone is just doing their own thing, and even then, there’s no collaboration or unity to discuss cases together, and this potentially leads to arguments, as sometimes one person takes on the responsibility of another.* (E5)

Given all these listed causes, many participating nurses realized that an authoritarian and imposing stance only increases and further exacerbates these conflicts:

*I don’t like being authoritarian, having to be forceful and say, “That’s how it is, period.” Because things just keep getting worse, it’s turning into a snowball effect. One thing leads to another, right?* (E2)

### Strategies for Managing Conflicts: Individual and Organizational

Multiple management approaches were identified, including individual ones such as respectful and harmonious dialogue, reflection and awareness of past mistakes, as well as monitoring whether there has been progress in relation to erroneous behaviors. Similarly, it was found that granting privacy and maintaining a cordial demeanor, that is, speaking in a low tone and making eye contact, allows the nurse to achieve a position of respect and authority.

*So, what I always try to do with all of them is look them in the eye, call them here to my office so we can talk. I have never, ever done that, and I think, I abhor those who do, of calling someone out in front of other people, being in a group, mentioning the name of someone who isn’t in the group, as an example.* (E12)

*I try to talk and look at both sides. I can’t remember anything specific that happened right now, but if there’s a conflict, I’ll listen to both sides and try to be as fair as possible, right? With each of them. Try to manage more fairly.* (E13)

*I think listening is fundamental, because often the act of venting can already calm things down, you know. And dialogue, you can’t have a good relationship or resolve conflict without it.* (E7)

*Observation and dialogue. And listening. The issue of communication itself. The best thing is interpersonal communication. It is respectful and obviously observe after a while whether it has improved.* (E8)

Therefore, other strategies, listed by the participants as essential for conflict management, are: the use of empathy; alignment meetings with the team; the use of shared and participatory decision-making with the technical team in defining schedules, distribution of functions, including therapeutic deliberation with the user;

*Holding group meetings, with RTs, where they share what they’re feeling, what’s needed in the sector, what’s happening, and what they think should be improved. So, everything is really related to dialogue. And one thing that I think is very important is for us to be involved in all the work there. Because to understand where that conflict, that anxiety, is coming from, you have to understand what the team member is going through with it.* (E10)

*Ah, yes, you have to have a lot of resilience, you have to have a lot of patience. Wow, that’s too much, because dealing with human beings is difficult. Isn’t it? So, you have to have a lot of patience, sometimes you have to think and rethink before speaking, have empathy, so as not to say a word that sometimes hurts the other person, sometimes it’s not even out of malice, but in the heat of the moment you end up saying what you shouldn’t.* (E13)

*Active listening is very important. You need to know how a person thinks, and why they think that way. What does she want? Don’t just let her pass the problem on to you to solve. I ask, how do you want me to solve this? How would you handle it if you were in my shoes? What would you do?* (E12)

Finally, a unique strategy adopted by one of the nurses was the use of prayer at the beginning of the shift, in an attempt to unite the team and promote respect, harmony, teamwork, and reinforce the focus on excellence in patient care.

*Another resource I always use is prayer, and I’ve included it in all shifts because during prayer we also talk about the importance of unity, helping colleagues, empathy, and staying together throughout the shift so as not to overburden anyone. So it’s a strategy to unite them and break down some of the tense atmosphere that sometimes arises from these conflicts.* (E11)

Organizational strategies provided by the hospital and the department’s management were mentioned in several interviews, such as online/in-person lectures and courses on nonviolent communication, moral and/or sexual harassment, team management, leadership, as well as webinars and platforms with various content related to the topic.

Furthermore, the sector promotes ongoing education on teamwork and a monthly general meeting consisting of the RT, the unit head, and a management council composed of one representative from each professional category, whose function is to bring the main demands of their group to the agenda.

*Last year, a course on nonviolent communication was held here at the entire hospital. It’s precisely so that everyone can develop this skill of resolving things through conversation, right? And not in a way that could be misinterpreted, or offend the other person, or cause them to feel offended. And it was a mandatory course. There’s always some course, some continuing education. We also have a platform for continuing education. So, everyone has access to the courses through their institutional email. There are several free courses you can take. It covers topics such as workplace harassment and sexual harassment.* (E2)

*She also called the occupational health team, and the psychologist came to talk to us a couple of times, both the occupational health nurse and the psychologist, because the team itself has been very tense for a while now due to the patients being very overwhelmed, and so everyone is taking a lot of sick leave.* (E7)

However, it was observed that none of these organizational strategies are specifically geared towards the topic of conflict management, leaving a gap in the professional training of these nurses.

*Several preparatory courses. I haven’t seen any about conflicts so far. Well, if there was one, I haven’t had the opportunity to do it yet; only communication, etc.* (E1)

Despite the above, it is important to emphasize that the participants indicated that conflict management has been developed over the years through two main approaches. The first one involved learning from other more experienced nurses and/or managers, who were recognized as experts in the field. The second way was through courses, updates, specializations/MBAs, since work structures change over the years, which directly impacts personal relationships and conflict management.

*I think it’s only when you experience it and have someone by your side who is good at managing conflicts that you truly understand. You’ll learn as you go, right? You learn from that person’s behavior. I learned from a few people. Primarily the leadership roles within the nursing division that I held when I was an RT.* (E9)

*These courses also help to... Not that you’ll have a ready-made recipe... They’ll give you this: just do this using formula A plus B, and you’ll get it. I don’t think that’s the right model, but they give you strategies, ways to deal with it, you know? How do you talk to an employee, how do you give feedback? So, that’s one way you can deal with it.* (E6)

Finally, it is important to highlight the role of training institutions in effectively developing and teaching the topic of conflict management to their future professionals in their training curricula. Paradoxically, participants reported having minimal exposure to the topic during their undergraduate studies, a fact that contributed to hindering their ability to resolve conflicts in their daily work practice.

## DISCUSSION

Conflict is defined as the real or perceived opposition of needs, values, and/or interests between two or more people, caused by individual or organizational factors, resulting in unwanted stress, tension, or negative feelings among the disputants^(11)^. Therefore, conflict management skills are an increasingly relevant topic in occupational contexts, especially in professions characterized by high emotional demands and intense human interaction, such as nursing, since nurses are exposed to conflicts daily.

Based on the results found, it was observed that nurses consider conflict management skills essential to their work practice, linking them to the ability to mediate situations that arise during their shift, whether with the technical team, multidisciplinary team, or patients and their companions. In addition, many emphasized that it is also up to them to mitigate the situation so that it does not need to reach higher levels of hospital management or take on unexpected proportions. Therefore, mastering conflict management strategies becomes vital for nurses^(12)^.

Therefore, as the findings of this study indicate, conflict management should not be guided by the attempt to avoid it at all costs, but rather by the ability to recognize it as a phenomenon inherent in human relations and, above all, as an opportunity for organizational growth and improvement of collaborative practices. Conflict, when managed well, can be a catalyst for positive change, promoting innovation, process review, and the strengthening of interpersonal relationships^(13)^.

This aligns with what researchers have indicated: when conflicts involving nurses are poorly managed or ignored, such as when they are repetitive or when solutions are unsatisfactory, adverse consequences can arise, such as low productivity, negative work environments, increased stress and anxiety, poor patient care, increased absenteeism and turnover, deterioration of teamwork, job dissatisfaction, and damage to the institution’s reputation^(14)^.

Therefore, it is extremely important that nurses know how to handle these situations to prevent chaos from taking hold in the workplace, since they are in the position of supervisors of the technical team and a communication channel for the multidisciplinary team, as the patient remains under the direct care of nursing staff throughout their hospital stay.

According to those interviewed, the situations that cause the most conflict, specifically in the surgical ward, are work overload, the distribution of functions, team professionals with personal problems or more than one job, resistant caregivers, and a lack of interprofessional communication, especially that related to the medical team.

As researchers have found, the most common causes of conflict are individual differences, including variations in personality, values, perceptions, and work styles among team members. Furthermore, issues related to power and authority stand out, which can also trigger conflicts within an organization, including disputes about decision-making, distribution of responsibilities, organizational hierarchy, and ineffective communication^(2)^, which corroborates some of the results of the present study.

In this way, instead of being seen as an obstacle, conflict can reveal legitimate differences of opinion, values, or priorities among professionals, which, when discussed respectfully and in a structured manner, contribute to building stronger consensus and more balanced decisions. Therefore, creating safe spaces for dialogue, encouraging active listening, and developing communication skills that foster empathy and mutual understanding are essential measures.

Considering the various causes presented, it becomes necessary to highlight the different strategies listed for managing these conflicts. Among these, individual strategies stood out, such as promoting harmonious and respectful dialogue, explicitly acknowledging the error and recognizing the professionals who managed to overcome it, maintaining a discreet and cordial posture during conversations, and avoiding authoritarian and imposing attitudes as a means of resolution.

In this sense, it is evident that collaborative conflict resolution aligns with previously published evidence, which indicates that in high-pressure environments such as the healthcare sector, cooperation and open dialogue are crucial for effectively managing conflicts^(15,16)^.

Therefore, the participants highlighted the importance of promoting alignment meetings and adopting a democratic stance when deliberating and preparing work schedules, to make the technical team feel a sense of belonging to that work organization and not perceive any injustice or favoritism in the decisions and actions taken by the nurse manager. For this purpose, the use of participatory and democratic management techniques creates a balance between the interests of conflicting parties, helping nursing managers to deal with situations in which it is not possible to achieve full satisfaction for all involved^(16,17)^.

Furthermore, the nurses mentioned several organizational strategies that contributed to conflict management as useful, such as lectures, courses, webinars, continuing education, and meetings with the management board. Nevertheless, none of these initiatives were specifically aimed at the topic of conflict, highlighting a training gap in this area.

This is a concerning fact, considering the need to implement educational processes aimed at conflict resolution. Mastering negotiation and public speaking skills allows a more harmonious and productive work environment, as well as higher quality patient care^(18)^. Accordingly, the findings also corroborate the evidence from a study conducted with medical and nursing undergraduates, which highlighted simulation in hospital wards as a strategy for developing essential skills, including conflict negotiation^(19)^, considered vital for clinical practice.

It is therefore clear that institutional effort in this direction is essential, since healthcare organizations face a continuous shortage of personnel and increasing patient demands. Thus, equipping nurses with conflict management skills can help mitigate stress and reduce staff turnover^(15)^.

Additionally, when conflicts are resolved constructively, a more stable and motivating environment is established, in which professionals feel valued and engaged in their roles^(20)^,strengthening the hospital’s ability to attract and retain qualified talent, which is essential for the effective operation of healthcare units.

The recurrence of these themes in various studies suggests that nurses’ ability to manage conflicts effectively may depend not only on organizational factors, but also on individual characteristics^(21,22)^.

However, it is known that such characteristics are not innate, so professionals need to develop them throughout their careers, either through courses and specializations or through interaction with other more experienced nurses/managers, who, due to their experience, tend to perceive conflict situations more clearly and manage them more effectively^(23)^.

In this regard, it is important for educational institutions to invest in content that prepares students to deal with conflict, which was not observed in the present study, given that the nurses had little or no contact with the subject during their undergraduate studies. These data contribute to the results of other investigations that have shown that curricula remain predominantly focused on technical and scientific content, relegating so-called relational skills to a secondary role^(23,24)^.

In addition, the limited number of teachers with supplementary training in leadership, communication, and negotiation restricts the approach to this topic to theoretical discussions, without proper connection to practice. The hierarchical institutional culture, frequently present in universities and health services, also hinders the development of spaces for dialogue about real conflicts, reinforcing the invisibility of the issue^(24)^.

Added to this is the curricular overload and the lack of active methodologies, which could enable simulations, reflections, and concrete problem-solving strategies. Therefore, conflict management remains a gap in training, which can compromise the future professional’s performance in contexts of high relational and organizational complexity^(24)^, such as the surgical ward and the hospital environment in general.

The study presents limitations that must be understood in light of the logic of qualitative research, whose purpose is not the generalization of results, but rather the in-depth understanding of a phenomenon in its specific context. The participation of 13 nurses proved sufficient to achieve data significance, allowing the identification of patterns and singularities in the perception of conflict management. However, conducting the research in only one public hospital institution limits the diversity of organizational and cultural contexts.

Another significant limitation is the lack of a detailed characterization of the surgical ward. In this regard, it is important to emphasize the need for studies that contain detailed information about the ward’s description, including the number of beds, the profile of patients served, the physical structure, and the criteria for determining the size of the nursing staff.

Although participants pointed out gaps in academic training on the subject, the study did not delve into the analysis of undergraduate nursing curricula. Even though these limitations exist, they do not invalidate the results obtained, but rather indicate aspects that can be explored in subsequent studies, expanding the scope and applicability of the reflections developed here.

Identifying the factors associated with conflict management reveals that this skill is not limited to technical expertise, but involves ethical, emotional, and communicational dimensions, which are often neglected in training and professional practice.

Recognizing that nurses act as mediators in tense situations among teams, patients, and families reinforces the need to invest in training that develops skills such as active listening, empathy, assertiveness, and emotional intelligence. By highlighting these causes, the study helps to denaturalize conflicts as mere side effects of hospital routine, proposing a more critical and proactive approach that considers the institutional context and the power dynamics involved.

Therefore, this study contributes to broadening the understanding of conflict management as an essential competency in nursing practice, with direct implications for the quality of care, the professionals’ mental health, and health services efficiency. By promoting critical reflection on the challenges and possibilities of this competency, the article reinforces the importance of developing institutional policies, comprehensive training, and institutional management committed to collective well-being and the humanization of relationships in the hospital environment.

## CONCLUSION

The analysis of hospital nurses’ perceptions of conflict management as a professional competency reveals significant contributions to the fields of nursing and healthcare, especially regarding the appreciation of interpersonal skills and the creation of more collaborative and resilient work environments. The causes of the conflicts, in turn, reveal the complexity of interpersonal relationships in the hospital environment, marked by work overload, rigid hierarchies, communication failures, and divergences in values.

In this sense, having ongoing training on the topics of leadership and effective communication is essential for honing this skill and ensuring the sustainability of teams. The effectiveness of conflict management in the nursing environment depends on a combination of individual skills, practical experience, and organizational support. To improve the work environment, productivity, and service quality, it is essential to invest in educational processes, promote a culture of dialogue and cooperation, and develop mediation skills from academic training to ongoing professional practice.

Individual strategies, such as self-control and the pursuit of dialogue, are fundamental, but insufficient if they are not accompanied by institutional actions that promote an organizational culture based on transparency, mutual respect, and the appreciation of professionals. The implementation of participatory management policies, spaces for listening and conflict mediation, as well as continuous professional development programs, are examples of measures that can strengthen the role of nurses as agents of change.

Furthermore, the need for the development and deepening of disciplines during undergraduate studies that prepare future professionals effectively for complex work environments with significant interpersonal demands becomes clear.

## Data Availability

All the data supporting the results of this study were published in the article itself.
