# Supplementary material for: Conflict management as a nurse’s competency in the surgical ward
Source: Rev Esc Enferm USP. 2026 Mar 6;60:e20250426. doi: 10.1590/1980-220X-REEUSP-2025-0426en (PMC12965661; doi:10.1590/1980-220X-REEUSP-2025-0426en)
Supplement: Translation

# Translation: Gerenciamento de conflitos como competência do enfermeiro em enfermaria cirúrgica

**DOI:** 10.1590/1980-220X-REEUSP-2025-0426pt

**Authors:** Ana Laura Pircio, Laura Andrian Leal, Divanice Contim, Silvia Helena Henriques, Iasmin Gabrielli da Silva, Ivaneia Alves Pereira Sobrinho, Luan Gagossian Savóia, Silvio Luiz de Flório, Josué Souza Gleriano, Andrea Bernardes, Bethania Ferreira Goulart, Priscila Linardi Guimarães

**Affiliations:** 1 Universidade Federal do Triângulo Mineiro, Departamento de Enfermagem, Uberaba, MG, Brasil.; 2 Universidade de São Paulo, Escola de Enfermagem de Ribeirão Preto, Ribeirão Preto, SP, Brasil.; 3 Centro Universitário Barão de Mauá, Departamento de Psicologia, Ribeirão Preto, SP, Brasil.; 4 Universidade do Estado de Mato Grosso, Departamento de Enfermagem, Tangará da Serra, MT, Brasil.; 5 Universidade Federal do Triângulo Mineiro, Departamento de Enfermagem em Educação e Saúde Comunitária, Uberaba, MG, Brasil.; 6 Prefeitura Municipal de Sertãozinho, Sertãozinho, SP, Brasil.

**Keywords:** Dissidências e Disputas, Negociação, Enfermagem, Competência Profissional, Enfermagem Perioperatória

## Abstract

**Objetivo:**

Analisar a percepção de enfermeiros de uma enfermaria cirúrgica sobre o gerenciamento de conflitos como competência profissional e as estratégias de manejo.

**Método:**

Trata-se de um estudo exploratório e qualitativo. O cenário foi uma Instituição Hospitalar Pública de Minas Gerais. A população foi constituída por enfermeiros da enfermaria cirúrgica, com dados coletados entre junho e dezembro de 2024. Utilizou-se análise de conteúdo indutiva. O estudo foi aprovado pelo Comitê de ética da instituição.

**Resultados:**

Emergiram categorias: “Fatores associados à competência de gerenciamento de conflitos”, relacionados a habilidades interprofissionais, éticas e comunicacionais, “Causas dos conflitos”, atreladas a sobrecarga de trabalho e outras e “Estratégias de manejo”, como reuniões de equipe e diálogo respeitoso, que contribuem para a melhoria da qualidade do cuidado.

**Conclusão:**

Os enfermeiros reconhecem o gerenciamento de conflitos como uma competência essencial à sua prática profissional, articulando habilidades técnicas, emocionais e comunicacionais. As estratégias de manejo identificadas revelam esforços para promover ambientes de trabalho mais harmônicos e qualificar o cuidado prestado.

## INTRODUÇÃO

A singularidade das organizações hospitalares tem sido evidenciada pela prestação de assistência a usuários em condições progressivamente mais críticas, o que exige dos profissionais de saúde competências específicas para enfrentar as constantes inovações tecnológicas, as variadas condições de trabalho, as mudanças comportamentais e a crescente exigência dos usuários, ocasionando, por vezes, transformações em seu processo de trabalho^(1)^.

Entre essas competências, destaca-se a capacidade de identificar e abordar conflitos, uma vez que tais situações repercutem no bem-estar organizacional da equipe de saúde, por estarem intrinsecamente relacionadas à convivência entre indivíduos que possuem valores, crenças, formações e opiniões distintas^(2)^.

O conflito configura-se como um processo no qual um indivíduo, de modo consciente e intencional, emprega esforços para impedir as ações de outro, estabelecendo algum tipo de bloqueio que compromete a consecução de seus objetivos e interesses. Dissidências, disputas e conflitos no âmbito das equipes de saúde são frequentes e podem resultar na redução da produtividade profissional, impactando negativamente o cuidado e o tratamento oferecidos aos pacientes. Dessa forma, a apropriação de competências por parte dos profissionais tornase essencial^(3)^.

Sabe-se que o enfermeiro, no contexto hospitalar, desempenha múltiplas atribuições que se articulam tanto ao cuidado direto ao usuário quanto às funções de caráter gerencial^(4)^. Nessa perspectiva, as competências são compreendidas como um conjunto integrado de conhecimentos, habilidades, atitudes e valores. Especificamente no caso dos enfermeiros, tais competências têm sido relacionadas à capacidade de combinar informações, condutas e princípios, bem como habilidades técnicas, raciocínio clínico, negociação de conflitos e comunicação efetiva^(5)^.

Assim, o enfermeiro hospitalar precisa lidar com múltiplas demandas e tarefas caracterizadas por elevado grau de exigência e responsabilidade, as quais, dependendo de sua experiência, de seus conhecimentos científicos e de sua postura profissional, podem favorecer ou não o surgimento de relações conflituosas nesse contexto de trabalho.

Para tanto, torna-se essencial que o enfermeiro desenvolva aptidões voltadas ao gerenciamento de conflitos, com vistas a assegurar a qualidade da assistência prestada e a manutenção da saúde organizacional. No desenvolvimento do processo de trabalho, é imprescindível que o enfermeiro demonstre proficiência em comunicação, observação, escuta ativa, senso crítico e empatia, a fim de entrever todas as faces de uma dissidência^(6)^.

Portanto, observa-se que, em setores complexos como as enfermarias cirúrgicas, essa temática configura uma problemática substancial que impacta diretamente a qualidade do cuidado, em virtude da complexidade inerente ao ambiente, caracterizado por alta rotatividade de pacientes, demandas assistenciais intensivas e necessidade de articulação contínua entre diferentes especialidades.

Nessa esfera ocorre um cuidado direto e ininterrupto prestado pela equipe de enfermagem, o que exige dos enfermeiros não apenas competências técnicas, mas também competências gerenciais e relacionais para lidar com situações adversas, frequentemente intensificadas pela pressão por resultados imediatos, escassez de recursos humanos e sobrecarga de trabalho^(7)^.

A enfermaria cirúrgica desempenha uma função predominantemente assistencial e operacional, direcionada ao acompanhamento clínico dos pacientes nos períodos pré e pós-operatório, sendo, em geral, organizada de acordo com a especialidade ou o tipo de procedimento cirúrgico. Por se tratar de uma área mais restrita^(7)^, observa-se uma lacuna de evidências científicas que problematizem sua dinâmica e ofereçam estratégias para seu adequado manejo^(4,5)^.

Portanto, investigar a percepção dos enfermeiros que atuam especificamente na enfermaria cirúrgica permite compreender com maior profundidade os desafios e estratégias empregadas no enfrentamento de conflitos neste contexto, contribuindo para o aprimoramento da formação profissional e para o desenvolvimento de políticas institucionais voltadas à promoção de ambientes de trabalho mais colaborativos e saudáveis, inclusive em outros setores.

Desta forma, este estudo apresenta os seguintes questionamentos: O enfermeiro percebe a negociação de conflitos como competência profissional? Como se dá a negociação de conflitos realizada pelo enfermeiro no contexto da enfermaria cirúrgica? Existem estratégias para manejo?

Ao compenetrar em um setor hospitalar, como a enfermaria cirúrgica, o estudo debruça-se sobre um ambiente onde os conflitos são não apenas frequentes, mas potencialmente mais intensos, dada a diversidade de profissionais envolvidos, a pressão por resultados imediatos e o cuidado com pacientes em situações críticas^(7)^. Essa escolha permite apreender nuances que poderiam passar despercebidas em contextos menos dinâmicos, revelando como os enfermeiros lidam com tensões que afetam diretamente a qualidade do cuidado, a segurança do paciente e o clima organizacional.

Nessa perspectiva, esta investigação possibilitará benefícios de forma iminente na qualidade do cuidado de enfermagem, haja vista as possibilidades de refletir sobre o tema e elencar possibilidades que resultem em uma atmosfera organizacional positiva.

Ademais, sua justificativa repousa no fato de tratar-se de uma temática premente, que demanda aprofundamento por parte dos profissionais da área, visto que o contexto hospitalar revela situações complexas nas quais a capacidade de negociar conflitos pode resultar em desfechos positivos tanto para o cuidado oferecido ao usuário quanto para a instituição.

Assim, o objetivo deste estudo foi analisar a percepção de enfermeiros de uma enfermaria cirúrgica sobre o gerenciamento de conflitos como competência profissional e as estratégias para manejo.

## MÉTODO

### Tipo do Estudo

Trata-se de um estudo do tipo exploratório, com abordagem qualitativa dos dados. O estudo qualitativo possibilita investigar percepções, comportamentos, culturas e relações sociais, permitindo uma compreensão holística e detalhada das realidades vividas pelos participantes^(8)^. O artigo foi preparado de acordo com as diretrizes do *Consolidated Criteria for Reporting Qualitative Research* (COREQ)^(9)^.

### Cenário do Estudo

O cenário do estudo foi uma Instituição Hospitalar Pública do Estado de Minas Gerais. O hospital selecionado é referência no Sistema Único de Saúde (SUS) em âmbito Municipal, Estadual e Nacional, que abrange o atendimento a pacientes que demandam cuidados de alta densidade tecnológica. Em específico foi selecionada a enfermaria cirúrgica, o que ocorreu de maneira aleatória.

### População e Critérios de Seleção

A população do estudo foi constituída por enfermeiros da enfermaria cirúrgica do hospital selecionado. O motivo de escolha da população deste setor refere-se ao fato de abarcar uma variedade de categorias profissionais atuando conjuntamente em prol do paciente. Nesse contexto, os enfermeiros assumem papel central na coordenação do cuidado, na supervisão da equipe técnica e na mediação das relações interpessoais, o que os torna particularmente expostos a situações de conflitos.

Foram incluídos e convidados de forma voluntária todos os enfermeiros atuantes da enfermaria cirúrgica, que possuíam o mínimo de seis meses de experiência, ao considerar que esse profissional possa ter lidado e manejado com questões de conflito no ambiente de trabalho. Não foram incluídos os profissionais que tinham menos que seis meses de experiência e/ou que estavam apenas em regime de cobertura eventual do setor, por não integrarem de forma permanente a equipe da enfermaria cirúrgica. Cabe salientar que o setor contava com 17 enfermeiros.

### Coleta de Dados

Ressalta-se que os enfermeiros foram convidados pessoalmente por carta convite pela primeira autora do estudo, que não exercia nenhuma influência de poder sobre os participantes recrutados. Foram informados os benefícios da pesquisa aos convidados e a participação de forma voluntária.

Para a coleta de dados, foram realizadas entrevistas semiestruturadas individuais, sendo o roteiro composto por duas partes: A primeira contendo dados sociodemográficos dos enfermeiros e a segunda contendo as seguintes questões: A negociação de conflitos é competência profissional do enfermeiro? Fale sobre e dê exemplos de situações; Como você realiza a negociação de conflitos no contexto da enfermaria cirúrgica? Reflita; Você teve alguma preparação para lidar com essas questões?

As entrevistas tiveram duração média de 15 minutos e foram realizadas pela primeira autora, que foi previamente capacitada pela orientadora; ocorreram individualmente no horário de intervalo dos enfermeiros no ambiente de trabalho, conforme a disponibilidade dos participantes. A coleta de dados ocorreu de junho a dezembro de 2024.

Antes do início da entrevista, foi feita uma explanação sobre o tema de investigação, os objetivos e a relevância de sua adesão à pesquisa, além da garantia do anonimato, buscando amenizar a preocupação de qualquer exposição futura. Ademais, as entrevistas foram audiogravadas e posteriormente manualmente transcritas, sem auxílio de software.

### Análise e Tratamento dos Dados

O corpus da avaliação foi composto pelo material transcrito das gravações, sendo utilizado o método de análise de conteúdo indutiva, com uso da modalidade da análise temática, sustentada pelo referencial teórico^(2,3)^.

Assim, foram consideradas as fases de interpretação e descrição dos dados deste estudo, tais como: familiarizar-se com os seus dados, gerar códigos iniciais, busca por temas, revisão de temas, definir e nomear temas, bem como produzir o relatório de análise^(10)^. Desta forma, foi realizada uma análise das falas a partir das gravações e os dados foram organizados em categorias para melhor discorrer sobre o assunto.

É notório enfatizar que os participantes foram identificados pela letra E de enfermeiro, e receberam uma numeração em algarismo arábico sequencial, garantindo-se, assim, o anonimato das falas. Desse modo, foram referenciados como E1, E2 e assim sucessivamente. Não houve uso de Inteligência Artificial.

### Aspectos Éticos

O estudo foi enviado e aprovado pelo Comitê de Ética em Pesquisa do Hospital selecionado da Universidade Federal do Triângulo Mineiro (HC-UFTM), segundo o número de parecer 6.931.316 de 2024.

## RESULTADOS

Participaram do estudo 13 enfermeiros, com predominância do sexo feminino, 12 (92,3%), com idades variando de 27 a 45 anos, sendo a média de 36,8 anos. Em relação à procedência, a maioria, sete (53,8%), era do estado de Minas Gerais, seguido da Bahia, dois (15,3%), São Paulo, dois (15,3%), Pernambuco, um (7,7%), e Maranhão, um (7,7%). O tempo de atuação dos participantes no setor de enfermaria cirúrgica variou de seis meses a nove anos, com média de 2,7 anos.

O tempo de formação foi de seis meses a 20 anos, com uma média de 11,7 anos. Em relação à atuação em outros setores do hospital, sete (53,8%) referem não ter atuado e seis (46,1%) referem ter atuado em outros setores, como neurologia, ortopedia, pediatria, pronto socorro adulto e infantil e Unidade de Terapia Intensiva (UTI) coronária, adulto e neonatal.

Destaca-se que, de todos os enfermeiros participantes, 11 (84,61%) possuíam especialização Lato sensu em variadas áreas, como Enfermagem Cirúrgica, Urgência e Emergência, Cardiologia, Terapia Intensiva e outros; além disso, cabe ressaltar que muitos profissionais tinham mais de uma especialização.

A partir dos dados das entrevistas, foram elencadas categorias para organização dos resultados, sendo: “Fatores Associados à Competência de Gerenciamento de Conflitos”; “Causas dos Conflitos” e “Estratégias para manejo de conflitos: individuais e organizacionais”.

### Fatores Associados À Competência de Gerenciamento de Conflitos

Nessa categoria, cabe contextualizar que os enfermeiros participantes referiram que lidam diariamente com uma multiplicidade de demandas e vínculos interpessoais relacionadas à equipe técnica subordinada, os pacientes com seus acompanhantes e todos os outros profissionais que compõem a equipe multiprofissional, pois recai sobre sua responsabilidade todos os cuidados à saúde que transcorrem no setor, sendo assim é incumbido a ele a função de liderar e gerir todas as situações.

Desse modo, os enfermeiros descreveram a competência de gerenciamento de conflitos como a capacidade de mediar, manejar, lidar, amenizar, resolver as situações adversas que aparecem durante o plantão. Outros fatores associados mencionados foram a necessidade da construção de um relacionamento interpessoal e interprofissional com imparcialidade e comunicação assertiva, conforme evidenciado nos relatos abaixo:

*Gerenciamento de conflitos, eu penso que é a forma como você lida dentro do seu trabalho, com as disputas que vão aparecendo, é tentar suavizar, seja com os seus próprios pares, seja com os técnicos de enfermagem, seja com outros profissionais.* (E6)

*É a gente saber lidar com as situações diárias, geralmente entre paciente, com os profissionais e entre os profissionais. A gente tem que ter ali um jogo de cintura, ter um bom relacionamento tanto interpessoal, quanto com a equipe, quanto um relacionamento bom com paciente, acompanhante e todos os profissionais do hospital, e sobretudo ter comunicação clara e ser imparcial, não podemos ter um lado ou preferência e sim saber analisar a situação.* (E7)

A capacidade de identificar e conceituar o conflito, bem como compreender que seu gerenciamento constitui uma competência essencial para o enfermeiro hospitalar, mostra-se imprescindível, uma vez que previne a deterioração das relações pessoais e reduz a necessidade de acessar instâncias superiores da gerência hospitalar.

*Não tem como evitar que aconteça, mas esse gerenciamento, na minha opinião, implica em conseguir pelo menos... Como é que eu posso dizer? Não deixar ficar pior, não deixar aumentar esse conflito, levar ele para frente, para um longo prazo. Tentar resolver ali da forma mais pontual possível.* (E2)

*Você precisa gerenciar pra aquilo não se tornar algo maior, pra aquilo não adquirir outras proporções. Eu acho que a gerenciamento do conflito é isso, é você tentar resolver de forma mais, vamos dizer assim, rápida, pra você não ficar criando em cima de um conflito, outros conflitos ou outras situações. Eu acho que seria isso.* (E1)

### Causas dos Conflitos

Foram citadas as causas mais comuns relacionadas ao surgimento de dissidências/conflitos no setor da enfermaria cirúrgica, sendo a principal a sobrecarga de trabalho, em decorrência de absenteísmos e atestados. Contudo, a equipe noturna refere ter, ainda, um fator agravante que corrobora essa sobrecarga, uma vez que no seu período de trabalho não conta com a presença de acadêmicos, residentes, médicos suficientes e até a própria Responsável Técnica (RT) do setor.

Ademais, outras causas de conflitos descritas foram relacionadas à equipe técnica ter uma perspectiva de injustiça em relação às funções delegadas e um relacionamento interpessoal desarmônico. Além disso, os participantes também relataram que, quando profissionais da equipe possuem problemas pessoais ou duplo vínculo empregatício, chegam ao trabalho cansados e estressados, o que frequentemente resulta na transferência dessa tensão para os colegas ou pacientes. Assim, esses profissionais passam a agir de forma desrespeitosa, mal-educada, explosiva, além de, por vezes, ignorarem as demandas dos acompanhantes, o que contribui para aumento de atritos e desavenças.

Outrossim, acompanhantes não colaborativos também foram citados como potencializadores de conflitos. E, por fim, outra causa em relação à equipe multiprofissional é a falta de cuidado interprofissional, uma vez que os cuidados fragmentados e não cooperantes geram falhas na comunicação, principalmente entre médicos e enfermeiros, conforme explícito nos depoimentos:

*Algo que envolve muito gerenciamento de conflito é a divisão, a divisão de enfermagem. É uma das coisas que eu mais venho percebendo, por falta de profissionais. Então, muitas vezes a gente precisa, né, ter uma organização que acaba não sendo da forma que seria na legislação, essa divisão, porque a gente não tem os profissionais suficientes.* (E4)

*Eu acho que a noite tem mais conflito que o dia. De dia tem mais gente para ajudar, tem mais profissionais. Chega o médico, ele fala tudo. Chega o acadêmico, fala tudo. Chega o residente, fala tudo. Chega o psicólogo, chega... Então, são tantos profissionais que acaba que não sobrecarrega tanto a equipe de reclamações. Agora a noite é só a gente que escuta. Quanto mais for lá, mais eles reclamam. Aí não tem como chamar o médico, o residente para essas situações. Aí a equipe fica mais cansada, mais sobrecarregada.* (E3)

*Às vezes o técnico que trabalha em vários lugares, já chega estressado, e ainda com um problema familiar e vai descontar no paciente.* (E11)

*São 7 técnicos de enfermagem pra 44, 45 pacientes, é muito pouco. É muito pouca equipe pra muito paciente. Então não tem jeito. Nem que eu queira, eu não consigo nem pôr um técnico em cada enfermaria.* (E9)

*Como tem muita sobrecarga vejo que cada um só faz o seu e olhe lá, não tem colaboração ou união para discutir casos em conjunto e isso potencialmente leva a discussão, pois as vezes um entra na função do outro.* (E5)

Diante de todas essas causas listadas, muitos enfermeiros participantes perceberam que uma postura autoritária e impositiva só aumenta e hostiliza ainda mais esses atritos:

*Eu não gosto de ser autoritária, ter que ser impositiva e falar, é assim, ponto final. Porque as coisas vão só piorando, vai virando uma bola de neve. Uma coisa puxa a outra, né?* (E2)

### Estratégias Para Manejo de Conflitos: Individuais e Organizacionais

Constataram-se múltiplas modalidades de manejo, como as individuais, a exemplo do diálogo respeitoso e harmônico, reflexão e conscientização sobre falhas cometidas, bem como monitorar se houve uma progressão em relação aos comportamentos equivocados. Similarmente, verificou-se que conceder privacidade e zelar por uma postura cordial, ou seja, falar em tom baixo, olhando no olho, faz com que o enfermeiro consiga galgar a uma posição de respeito e autoridade:

*Então, o que eu tento fazer sempre com todos eles é olhar nos olhos, chamar aqui na minha sala pra gente conversar. Nunca, nunca fiz isso e acho, abomino quem faz, de chamar atenção na frente das outras pessoas, de estar em um grupo reunido, de citar nome de quem não tá no grupo, como exemplo.* (E12)

*Eu tento conversar e olhar pra ambos os lados. É que eu não tô pensando aqui, agora, numa coisa específica que aconteceu, mas se tiver um conflito eu vou ouvir as duas partes e eu vou tentar ser mais justa possível, né? Com cada um deles. Tentar gerenciar de forma mais justa.* (E13)

*Acho que ouvir é fundamental, porque muitas vezes o fato de a pessoa desabafar, ela já dá uma acalmada ali, né. E o diálogo, não tem como ter um bom relacionamento, resolver conflito sem diálogo.* (E7)

*Observação e diálogo. E escuta. A questão da comunicação em si. A melhor coisa é comunicação interpessoal. E respeitosa e claro observar depois de um tempo se melhorou.* (E8)

Por conseguinte, outras estratégias, elencadas pelos participantes como essenciais para o gerenciamento de conflitos, são: uso da empatia; reunião de alinhamento com a equipe; utilização da tomada de decisão compartilhada e participativa com a equipe técnica na definição de escalas, distribuição de funções, inclusive deliberação terapêutica para com o usuário:

*Fazer reuniões em grupo mesmo, com RT, e eles lançam o que eles estão sentindo, o que está precisando no setor, o que está acontecendo, o que eles acham que deve melhorar. Então, tudo é relacionado ao diálogo mesmo. E uma coisa que eu acho muito importante, é a gente estar envolvida em todo o trabalho ali dentro. Porque para você entender de onde está vindo aquele conflito, aquela ansiedade, você tem que entender o que o membro da equipe está passando com aquilo.* (E10)

*Ah, sim, tem que ter muita resiliência, tem que ter muita paciência. Nossa, demais, porque lidar com o ser humano é difícil. Né? Então, tem que ter muita paciência, às vezes a gente tem que pensar e repensar antes de falar, ter empatia, pra não falar uma palavra que às vezes machuca o outro, às vezes nem é por mal, mas ali no calor do momento acaba falando o que não devia.* (E13)

*Escuta ativa é muito importante. Você saber como a pessoa pensa, por que ela pensa daquele jeito. O que ela quer? Não deixar ela simplesmente jogar o problema pra você resolver. Eu pergunto, como você quer que eu resolva? Como você resolveria se você estivesse no meu lugar? O que você iria fazer?* (E12)

Por fim, uma estratégia peculiar adotada por uma das enfermeiras foi a utilização da oração no início do plantão, na tentativa de unir a equipe e promover o respeito, a harmonia, o trabalho em equipe e reforçar o foco na excelência do atendimento ao paciente.

*Outro recurso que eu uso sempre é o da oração e coloquei isso em todos os plantões, porque no momento da oração a gente usa para falar também a importância da união, de ajudar o colega, da empatia, de permanecer durante todo o plantão de mãos dadas, para não sobrecarregar ninguém, então é uma estratégia para unir eles e acabar quebrando um pouco do clima tenso que por vezes fica em decorrência desses conflitos.* (E11)

Estratégias organizacionais disponibilizadas pelo hospital e pela gerência do setor foram mencionadas em algumas entrevistas, como palestras e cursos online/presenciais sobre comunicação não violenta, assédio moral e/ou sexual, gerenciamento de equipe, liderança, além de webinar e plataformas com vários conteúdos que se aproximam ao tema.

Além disso, o setor promove educação permanente sobre trabalho em equipe e uma reunião geral mensal constituída pela RT, a chefe de unidade e um conselho gestor composto por um representante de cada categoria profissional, com a função de levar as principais demandas de seu grupo em pauta:

*Ano passado foi um curso pactuado aqui no hospital inteiro sobre comunicação não violenta. Justamente para todo mundo desenvolver essa habilidade de resolver as coisas conversando, né? E não de forma a ser mal interpretado ou de ofender o outro ou se sentir ofendido. E era um curso obrigatório. Sempre tem algum curso, alguma educação permanente. Tem também a plataforma de educação permanente. Então, todo mundo tem acesso pelo e-mail institucional aos cursos. Tem vários cursos gratuitos que você pode fazer. Tem sobre assédio moral, sobre assédio sexual.* (E2)

*Já chamou também a equipe do trabalho, que vem a psicóloga conversar com a gente umas duas vezes, tanto o enfermeiro do trabalho quanto a psicóloga, porque a equipe mesmo tem tempo que está muito arisca por causa dos pacientes que estão muito pesados, e aí todo mundo coloca muito atestado.* (E7)

Contudo, foi observado que nenhuma dessas estratégias organizacionais é voltada para o tema em específico de gerenciamento de conflito, deixando uma lacuna na capacitação profissional desses enfermeiros.

*Vários cursinhos. De conflitos até agora eu não vi nenhum. Bom se teve eu ainda não tive a oportunidade de fazer; só de comunicação etc.* (E1)

Apesar do exposto, é importante frisar que os participantes elencaram que o manejo de conflito foi desenvolvido ao longo dos anos por duas vias principais. A primeira consistiu na aprendizagem com outros enfermeiros e/ou gestores mais experientes, destacados como referências no assunto. A segunda, foi por meio de cursos, atualizações, especializações/MBA, já que as estruturas de trabalho vão se alterando ao longo dos anos, o que impacta diretamente nas relações pessoais e de gerenciamento de conflito:

*Eu acho que só quando a gente vivencia e tem alguém do seu lado que é bom em gerenciar conflitos. Você vai aprendendo, né? Você vai aprendendo com a conduta daquela pessoa. Eu aprendi com algumas pessoas. Principalmente chefias da divisão de enfermagem que eu tive quando eu era RT.* (E9)

*Esses cursos, eles ajudam também a... Não que você vai ter uma receita pronta... Eles vão te fornecer, assim, ó, faz isso na fórmula A mais B e que você vai conseguir. Eu não penso que é esse modelo, mas eles te dão estratégias, formas de como lidar com isso, né? Como é que você conversa, como é que você dá um feedback para um funcionário. Então, isso é uma forma de você lidar.* (E6)

Por fim, é importante ressaltar o papel das instituições formadoras para efetivamente desenvolver e ensinar seus futuros profissionais a temática do gerenciamento de conflitos em seus currículos formativos. Contraditoriamente, os participantes referiram que tiveram mínimo contato com a temática durante a graduação, fato que contribuiu para dificultar o desempenho na resolução de conflitos no dia a dia da prática laboral.

## DISCUSSÃO

Conflito é definido como a oposição real ou percebida de necessidades, valores e/ou interesses entre duas ou mais pessoas, que é causado por fatores individuais ou organizacionais, resultando em estresse indesejado, tensão ou sentimentos negativos entre os disputantes^(11)^. Desse modo, a competência de gerenciamento de conflitos é um tema cada vez mais relevante em contextos ocupacionais, especialmente em profissões caracterizadas por altas demandas emocionais e de intensa interação humana, como a enfermagem, uma vez que eles estão expostos a conflitos diariamente.

Mediante os resultados encontrados, foi constatado que os enfermeiros consideram a competência de gerenciamento de conflitos essencial para a prática de trabalho, sendo atrelada à capacidade de mediação de uma situação que surge durante seu plantão, seja com a equipe técnica, multiprofissional ou pacientes e acompanhantes. Além disso, muitos enfatizaram que cabe a eles também amenizar a situação para que ela não precise chegar a instâncias maiores de gestão do hospital ou tome proporções inesperadas. Portanto, dominar estratégias de gerenciamento de conflitos torna-se vital para enfermeiros^(12)^.

Destarte, como apontam os achados deste estudo, o gerenciamento de conflitos não deve ser orientado pela tentativa de evitá-lo a todo custo, mas sim pela capacidade de reconhecê-lo como um fenômeno inerente às relações humanas e, sobretudo, como uma oportunidade de crescimento organizacional e aprimoramento das práticas colaborativas. O conflito, quando bem conduzido, pode ser um catalisador de mudanças positivas, promovendo inovação, revisão de processos e fortalecimento das relações interpessoais^(13)^.

Isso vai ao encontro do que pesquisadores apontam, ou seja, quando conflitos envolvendo enfermeiros são mal gerenciados ou ignorados, como quando são repetitivos ou quando as soluções são insatisfatórias, podem surgir adversidades como baixa produtividade, ambientes de trabalho negativos, aumento do estresse e da ansiedade, atendimento precário ao paciente, aumento do absenteísmo e da rotatividade, deterioração do trabalho em equipe, insatisfação no trabalho e danos à reputação da instituição^(14)^.

Por isso, é de extrema importância que os enfermeiros saibam lidar com essas situações, a fim de evitar que o caos se instale no ambiente laboral, uma vez que eles estão na posição de supervisores da equipe técnica e canal de comunicação para a equipe multiprofissional, já que o paciente fica todo o tempo de internação diretamente sob os cuidados de enfermagem.

Segundo os entrevistados, as situações que mais causam conflitos, na enfermaria cirúrgica, em específico, são a sobrecarga de trabalho, a distribuição de funções, profissionais da equipe com problemas pessoais ou mais de um vínculo laboral, acompanhantes resistentes e escassez de comunicação interprofissional, principalmente aquela relacionada à equipe médica.

Conforme é vislumbrado por pesquisadores, as causas mais comuns de conflito são as diferenças individuais que incluem variações de personalidade, valores, percepções e estilos de trabalho entre os membros da equipe. Além disso, destacamse questões relacionadas ao poder e à autoridade, que também podem desencadear conflitos dentro de uma organização, incluindo disputas acerca da tomada de decisão, distribuição de responsabilidades, hierarquia organizacional e comunicação ineficaz^(2)^, o que corrobora parte dos resultados do presente estudo.

Desta forma, ao invés de ser encarado como um obstáculo, o conflito pode revelar divergências legítimas de opinião, valores ou prioridades entre os profissionais, que, quando discutidas de forma respeitosa e estruturada, contribuem para a construção de consensos mais sólidos e decisões mais equilibradas. Portanto, criar espaços seguros para o diálogo, estimular a escuta ativa e desenvolver competências comunicacionais que favoreçam a empatia e o entendimento mútuo são medidas primordiais.

Considerando as diversas causas expostas, torna-se necessário salientar as diferentes estratégias elencadas para o manejo desses conflitos. Entre elas, destacaram-se estratégias de cunho individual, tais como a promoção de um diálogo harmônico e respeitoso, a explicitação do erro acompanhada do reconhecimento dos profissionais que conseguiram superá-lo, a manutenção de uma postura discreta e cordial nos momentos de conversa e a evitar atitudes autoritárias e impositivas como meio de resolução.

Nesse sentido, fica evidente que a resolução colaborativa está alinhada às evidências já publicadas, as quais indicam que, em ambientes de alta pressão, como o setor da saúde, a cooperação e o diálogo aberto são cruciais para lidar efetivamente com conflitos^(15,16)^.

Destarte, os participantes evidenciaram a importância de promover reuniões de alinhamento e adotar uma postura democrática no momento das deliberações e elaborações de escalas de trabalho, a fim de fazer com que a equipe técnica se sinta pertencente àquela organização laboral e não disponha de uma percepção de injustiça ou favoritismo sob as decisões e ações tomadas pelo enfermeiro gestor. Para tanto, a utilização do recurso de gestão participativa e democrática faz com que haja um equilíbrio entre os interesses das partes conflitantes, auxiliando os gerentes de enfermagem a lidarem com situações nas quais não é possível alcançar uma satisfação plena para todos os envolvidos^(16,17)^.

Além disso, os enfermeiros referiram como úteis algumas estratégias organizacionais que contribuíram para o gerenciamento de conflitos, como palestras, cursos, webinars, educação permanente e reuniões com o conselho gestor. Contudo, nenhuma dessas iniciativas estava especificamente direcionada à temática dos conflitos, ressaltando uma lacuna formativa nessa área.

Tal fato é preocupante, considerando a necessidade de implementar processos educativos voltados à resolução de conflitos. O domínio de técnicas de negociação e oratória possibilita um ambiente de trabalho mais harmônico, produtivo e uma assistência ao paciente de maior qualidade^(18)^. Destarte, os achados também corroboram as evidências de um estudo desenvolvido com graduandos da medicina e enfermagem, no qual se destacou a simulação em enfermarias como estratégia para o desenvolvimento de competências essenciais, entre elas a negociação de conflitos^(19)^, considerada vital para a prática clínica.

Nota-se, portanto, que é imprescindível o esforço institucional nessa direção, uma vez que as organizações de saúde enfrentam escassez contínua de pessoal e demandas crescentes de pacientes. Assim, equipar os enfermeiros com competências de gerenciamento de conflitos pode contribuir para mitigar o estresse e reduzir a rotatividade profissional^(15)^.

Além disso, quando os conflitos são solucionados de maneira construtiva, estabelece-se um ambiente mais estável e motivador, no qual os profissionais se sentem valorizados e engajados em seus papéis^(20)^, fortalecendo a capacidade do hospital de atrair e reter talentos qualificados, essenciais para o funcionamento eficaz das unidades de saúde.

A recorrência desses temas em diversos estudos sugere que a capacidade dos enfermeiros de gerenciar conflitos de forma eficaz pode depender não apenas de fatores organizacionais, mas também de características individuais^(21,22)^.

Entretanto, sabe-se que tais características não são inatas, de modo que os profissionais precisaram desenvolvê-las ao longo de sua carreira, seja por meio de cursos e especializações ou pela convivência com outros enfermeiros/gestores mais experientes, os quais, por sua vivência, tendem a perceber com maior clareza e a manejar com mais eficácia as situações de conflito^(23)^.

Nesse sentido, ressalta-se a importância de as instituições formadoras investirem em conteúdos que preparem o estudante para lidar com conflitos, o que não foi verificado no presente estudo, haja vista que os enfermeiros tiveram pouco ou nenhum contato com a temática durante a graduação. Esses dados contribuem para os resultados de outras investigações que evidenciaram que os currículos permanecem predominantemente centrados em conteúdos técnico-científicos, relegando as chamadas competências relacionais a um papel secundário^(23,24)^.

Além disso, o reduzido número de docentes com formação complementar em liderança, comunicação e negociação restringe a abordagem desse tema a discussões teóricas, sem a devida articulação com a prática. A cultura institucional hierárquica, frequentemente presente em universidades e serviços de saúde, também dificulta o desenvolvimento de espaços de diálogo sobre conflitos reais, reforçando a invisibilidade da temática^(24)^.

Soma-se a isso a sobrecarga curricular e a falta de metodologias ativas, que poderiam viabilizar simulações, reflexões e estratégias concretas de resolução. Dessa forma, o gerenciamento de conflitos permanece como uma lacuna na formação, o que pode comprometer a atuação do futuro profissional em contextos de elevada complexidade relacional e organizacional^(24)^, como a enfermaria cirúrgica e o ambiente hospitalar de modo geral.

O estudo apresenta limitações que devem ser compreendidas à luz da lógica da pesquisa qualitativa, cuja finalidade não é a generalização dos resultados, mas sim a compreensão aprofundada de um fenômeno em seu contexto específico. A participação de 13 enfermeiros revelou-se suficiente para alcançar a significância dos dados, permitindo identificar padrões e singularidades na percepção sobre o gerenciamento de conflitos. No entanto, a realização da pesquisa em apenas uma instituição hospitalar pública limita a diversidade de contextos organizacionais e culturais.

Outra limitação relevante é a ausência de uma caracterização detalhada da enfermaria cirúrgica. A este respeito, destaca-se a importância da elaboração de estudos que contenham informações detalhadas sobre a descrição da enfermaria, com o número de leitos, perfil dos pacientes atendidos, estrutura física e critérios de dimensionamento da equipe de enfermagem.

E, embora os participantes tenham apontado lacunas na formação acadêmica sobre o tema, o estudo não aprofundou a análise dos currículos dos cursos de graduação em enfermagem. Ainda que essas limitações estejam presentes, elas não invalidam os resultados obtidos, mas indicam aspectos que podem ser explorados em estudos subsequentes, ampliando o escopo e a aplicabilidade das reflexões aqui desenvolvidas.

A identificação dos fatores associados ao gerenciamento de conflitos evidencia que essa competência não se restringe ao domínio técnico, mas envolve dimensões éticas, emocionais e comunicacionais, frequentemente negligenciados na formação e na prática profissional.

Reconhecer que o enfermeiro atua como mediador em situações de tensão entre equipes, pacientes e familiares reforça a necessidade de investir em capacitações que desenvolvam habilidades como escuta ativa, empatia, assertividade e inteligência emocional. Ao trazer à tona essas causas, o estudo contribui para desnaturalizar os conflitos como meros efeitos colaterais da rotina hospitalar, propondo uma abordagem mais crítica e propositiva que considera o contexto institucional e as dinâmicas de poder envolvidas.

Portanto, o estudo contribui para ampliar a compreensão do gerenciamento de conflitos como uma competência essencial à prática da enfermagem, com implicações diretas na qualidade do cuidado, na saúde mental dos profissionais e na eficiência dos serviços de saúde. Ao promover uma reflexão crítica sobre os desafios e possibilidades dessa competência, o artigo reforça a importância do desenvolvimento de políticas institucionais e de uma formação integral e de um gerenciamento institucional comprometido com o bem-estar coletivo e com a humanização das relações no ambiente hospitalar.

## CONCLUSÃO

A análise da percepção de enfermeiros hospitalares sobre o gerenciamento de conflitos como competência profissional revela contribuições significativas para o campo da enfermagem e da saúde, especialmente no que tange à valorização das habilidades interpessoais e à construção de ambientes de trabalho mais colaborativos e resilientes. As causas dos conflitos, por sua vez, revelam a complexidade das relações interpessoais no ambiente hospitalar, marcado por sobrecarga de trabalho, hierarquias rígidas, falhas de comunicação e divergências de valores.

Nesse sentido, ter uma formação que perdure sobre os temas de liderança e comunicação eficaz é primordial para a lapidação dessa competência e sustentabilidade das equipes. A eficácia do gerenciamento de conflitos no ambiente de enfermagem depende de uma combinação de habilidades individuais, experiência prática e apoio organizacional. Para melhorar o clima laboral, a produtividade e a qualidade do atendimento, é fundamental investir em processos educativos, promover cultura de diálogo e cooperação, e desenvolver competências de mediação desde a formação acadêmica até a prática profissional contínua.

Estratégias individuais, como o autocontrole e a busca por diálogo, são fundamentais, mas insuficientes se não forem acompanhadas por ações institucionais que promovam uma cultura organizacional pautada na transparência, no respeito mútuo e na valorização dos profissionais. A implementação de políticas de gerenciamento participativa, espaços de escuta e mediação de conflitos, bem como programas de desenvolvimento profissional contínuo, são exemplos de medidas que podem fortalecer a atuação dos enfermeiros como agentes de mudança.

Além disso, fica explícita a necessidade do desenvolvimento e aprofundamento de disciplinas durante a graduação que pre-parem os futuros profissionais de forma assertiva para cenários laborais complexos com grandes exigências interpessoais.

## Data Availability

Todo o conjunto de dados que dá suporte aos resultados deste estudo foi publicado no próprio artigo.

# Translation: La gestión de conflictos como competencia del enfermero en el servicio quirúrgico

**DOI:** 10.1590/1980-220X-REEUSP-2025-0426es

**Authors:** Ana Laura Pircio, Laura Andrian Leal, Divanice Contim, Silvia Helena Henriques, Iasmin Gabrielli da Silva, Ivaneia Alves Pereira Sobrinho, Luan Gagossian Savóia, Silvio Luiz de Flório, Josué Souza Gleriano, Andrea Bernardes, Bethania Ferreira Goulart, Priscila Linardi Guimarães

**Affiliations:** 1 Universidade Federal do Triângulo Mineiro, Departamento de Enfermagem, Uberaba, MG, Brasil.; 2 Universidade de São Paulo, Escola de Enfermagem de Ribeirão Preto, Ribeirão Preto, SP, Brasil.; 3 Centro Universitário Barão de Mauá, Departamento de Psicologia, Ribeirão Preto, SP, Brasil.; 4 Universidade do Estado de Mato Grosso, Departamento de Enfermagem, Tangará da Serra, MT, Brasil.; 5 Universidade Federal do Triângulo Mineiro, Departamento de Enfermagem em Educação e Saúde Comunitária, Uberaba, MG, Brasil.; 6 Prefeitura Municipal de Sertãozinho, Sertãozinho, SP, Brasil.

**Keywords:** Disentimientos y Disputas, Negociación, Enfermería, Competencia Profesional, Enfermería Perioperatoria

## Abstract

**Objetivo:**

Analizar la percepción de los enfermeros de un servicio quirúrgico respecto a la gestión de conflictos como competencia profesional y las estrategias para gestionarla.

**Método:**

Se trata de un estudio exploratorio y cualitativo. El escenario fue un hospital público de Minas Gerais. La población del estudio estuvo constituida por enfermeros del servicio de cirugía, con datos recolectados entre junio y diciembre de 2024. Se utilizó análisis de contenido inductivo. El estudio fue aprobado por el comité de ética de la institución.

**Resultados:**

Surgieron categorías: “Factores asociados a la competencia en gestión de conflictos”, relacionados con habilidades interprofesionales, éticas y de comunicación; “Causas de los conflictos”, vinculadas a la sobrecarga de trabajo y otros factores; y “Estrategias de gestión”, como reuniones de equipo y diálogo respetuoso, que contribuyen a mejorar la calidad de la atención.

**Conclusión:**

Los enfermeros reconocen la gestión de conflictos como una competencia esencial en su práctica profesional, combinando habilidades técnicas, emocionales y de comunicación. Las estrategias de gestión identificadas revelan esfuerzos para promover entornos de trabajo más armoniosos y mejorar la calidad de la atención brindada.

## INTRODUCCIÓN

La singularidad de las organizaciones hospitalarias se ha puesto de manifiesto en la prestación de asistencia a usuarios en condiciones cada vez más críticas, lo que exige a los profesionales de la salud competencias específicas para hacer frente a las constantes innovaciones tecnológicas, las diversas condiciones de trabajo, los cambios de comportamiento y las crecientes exigencias de los usuarios, lo que a veces provoca transformaciones en su proceso de trabajo^(1)^.

Entre estas competencias, destaca la capacidad de identificar y abordar los conflictos, ya que estas situaciones repercuten en el bienestar organizacional del equipo de salud, al estar intrínsecamente relacionadas con la convivencia entre individuos que tienen valores, creencias, formaciones y opiniones diferentes^(2)^.

El conflicto se configura como un proceso en el que un individuo, de manera consciente e intencionada, emplea esfuerzos para impedir las acciones de otro, estableciendo algún tipo de bloqueo que compromete la consecución de sus objetivos e intereses. Las disidencias, disputas y conflictos en el ámbito de los equipos de salud son frecuentes y pueden dar lugar a una reducción de la productividad profesional, lo que repercute negativamente en la atención y el tratamiento que se ofrece a los pacientes. Por lo tanto, la adquisición de competencias por parte de los profesionales se vuelve esencial^(3)^.

Es sabido que el enfermero, en el contexto hospitalario, desempeña múltiples funciones que se articulan tanto con la atención directa al usuario como con las funciones de carácter gerencial^(4)^. Desde esta perspectiva, las competencias se entienden como un conjunto integrado de conocimientos, habilidades, actitudes y valores. Específicamente en el caso de los enfermeros, estas competencias se han relacionado con la capacidad de combinar información, conductas y principios, así como habilidades técnicas, razonamiento clínico, negociación de conflictos y comunicación eficaz^(5)^.

Así, el enfermero del hospital debe hacer frente a múltiples demandas y tareas caracterizadas por un alto grado de exigencia y responsabilidad que, dependiendo de su experiencia, sus conocimientos científicos y su postura profesional, pueden favorecer o no la aparición de relaciones conflictivas en este contexto laboral.

Para ello, es esencial que el enfermero desarrolle habilidades orientadas a la gestión de conflictos, con el fin de garantizar la calidad de la asistencia prestada y el mantenimiento de la salud organizacional. En el desarrollo del proceso de trabajo, es imprescindible que el enfermero demuestre competencia en comunicación, observación, escucha activa, sentido crítico y empatía, con el fin de vislumbrar todas las facetas de una disidencia^(6)^.

Por lo tanto, se observa que, en sectores complejos como las enfermerías quirúrgicas, esta temática constituye un problema sustancial que impacta directamente en la calidad de la atención, debido a la complejidad inherente al entorno, caracterizado por una alta rotación de pacientes, demandas asistenciales intensivas y la necesidad de una articulación continua entre diferentes especialidades.

En este ámbito, el equipo de enfermería presta una atención directa e ininterrumpida, lo que exige a los enfermeros no solo competencias técnicas, sino también competencias gerenciales y relacionales para hacer frente a situaciones adversas, a menudo intensificadas por la presión por obtener resultados inmediatos, la escasez de recursos humanos y la sobrecarga de trabajo^(7)^.

La enfermería quirúrgica desempeña una función predominantemente asistencial y operativa, dirigida al seguimiento clínico de los pacientes en los periodos pre y posoperatorios, y suele estar organizada según la especialidad o el tipo de procedimiento quirúrgico. Al tratarse de un área más restringida^(7)^, se observa una laguna de evidencia científica que problematice su dinámica y ofrezca estrategias para su adecuado manejo^(4,5)^.

Por lo tanto, investigar la percepción de los enfermeros que trabajan específicamente en la enfermería quirúrgica permite comprender con mayor profundidad los retos y las estrategias empleadas para afrontar los conflictos en este contexto, contribuyendo a la mejora de la formación profesional y al desarrollo de políticas institucionales orientadas a la promoción de entornos de trabajo más colaborativos y saludables, incluso en otros sectores.

De este modo, este estudio plantea las siguientes preguntas: ¿Percibe el enfermero la negociación de conflictos como una competencia profesional? ¿Cómo se lleva a cabo la negociación de conflictos por parte del enfermero en el contexto de la enfermería quirúrgica? ¿Existen estrategias para su manejo?

Al centrarse en un sector hospitalario como la enfermería quirúrgica, el estudio se centra en un entorno en el que los conflictos no solo son frecuentes, sino potencialmente más intensos, dada la diversidad de profesionales involucrados, la presión por obtener resultados inmediatos y el cuidado de pacientes en situaciones críticas^(7)^. Esta elección permite captar matices que podrían pasar desapercibidos en contextos menos dinámicos, revelando cómo los enfermeros manejan las tensiones que afectan directamente la calidad de la atención, la seguridad del paciente y el clima organizacional.

Desde esta perspectiva, esta investigación permitirá obtener beneficios inmediatos en la calidad de la atención de enfermería, dadas las posibilidades de reflexionar sobre el tema y enumerar posibilidades que den lugar a un ambiente organizacional positivo.

Además, su justificación se basa en el hecho de que se trata de un tema urgente, que exige una profundización por parte de los profesionales del área, ya que el contexto hospitalario revela situaciones complejas en las que la capacidad de negociar conflictos puede dar lugar a resultados positivos tanto para la atención prestada al usuario como para la institución.

Así, el objetivo de este estudio fue analizar la percepción de los enfermeros de una sala quirúrgica sobre la gestión de conflictos como competencia profesional y las estrategias para su manejo.

## MÉTODO

### Tipo de Estudio

Se trata de un estudio de tipo exploratorio, con un enfoque cualitativo de los datos. El estudio cualitativo permite investigar percepciones, comportamientos, culturas y relaciones sociales, lo que permite una comprensión holística y detallada de las realidades vividas por los participantes^(8)^. El artículo se preparó de acuerdo con las directrices de los Criterios Consolidados para la Presentación de Informes de Investigación Cualitativa (*Consolidated Criteria for Reporting Qualitative Research* – COREQ)^(9)^.

### Escenario del Estudio

El escenario del estudio fue un hospital público del estado de Minas Gerais. El hospital seleccionado es una referencia en el Sistema Único de Salud (SUS) a nivel municipal, estatal y nacional, que abarca la atención a pacientes que requieren cuidados de alta densidad tecnológica. En concreto, se seleccionó la enfermería quirúrgica, lo que se hizo de forma aleatoria.

### Población y Criterios de Selección

La población del estudio estuvo constituida por enfermeros de la sala quirúrgica del hospital seleccionado. La razón para elegir la población de este sector se refiere al hecho de que abarca una variedad de categorías profesionales que trabajan conjuntamente en beneficio del paciente. En este contexto, los enfermeros asumen un papel central en la coordinación de la atención, la supervisión del equipo técnico y la mediación de las relaciones interpersonales, lo que los hace particularmente expuestos a situaciones de conflicto.

Se incluyeron e invitaron de forma voluntaria a todos los enfermeros que trabajaban en la enfermería quirúrgica y que tenían un mínimo de seis meses de experiencia, al considerar que estos profesionales pueden haber lidiado y manejado cuestiones de conflicto en el entorno laboral. No se incluyó a los profesionales con menos de seis meses de experiencia y/o que solo estaban cubriendo temporalmente el sector, ya que no formaban parte del equipo permanente de la enfermería quirúrgica. Cabe señalar que el sector contaba con 17 enfermeros.

### Recolección de Datos

Cabe destacar que los enfermeros fueron invitados personalmente mediante una carta de invitación por la primera autora del estudio, que no ejercía ninguna influencia de poder sobre los participantes reclutados. Se informó a los invitados de los beneficios de la investigación y de que la participación era voluntaria.

Para la recolección de datos, se realizaron entrevistas semiestructuradas individuales, con un guion compuesto por dos partes: la primera contenía datos sociodemográficos de los enfermeros y la segunda contenía las siguientes preguntas: ¿La negociación de conflictos es una competencia profesional del enfermero? Hable sobre ello y dé ejemplos de situaciones; ¿Cómo lleva a cabo la negociación de conflictos en el contexto de la enfermería quirúrgica? Reflexione; ¿Ha recibido alguna preparación para lidiar con estas cuestiones?

Las entrevistas tuvieron una duración media de 15 minutos y fueron realizadas por la primera autora, que había sido previamente capacitada por la orientadora; se llevaron a cabo de forma individual durante el descanso de los enfermeros en el lugar de trabajo, según la disponibilidad de los participantes. La recolección de datos se realizó entre junio y diciembre de 2024.

Antes de comenzar la entrevista, se explicó el tema de la investigación, los objetivos y la relevancia de su participación en la misma, además de garantizar el anonimato, con el fin de mitigar cualquier preocupación por una futura exposición. Además, las entrevistas fueron grabadas y posteriormente transcritas manualmente, sin ayuda de software.

### Análisis y Tratamiento de los Datos

El corpus de la evaluación se compuso del material transcrito de las grabaciones, utilizando el método de análisis de contenido inductivo, con el uso de la modalidad de análisis temático, respaldado por el marco teórico^(2,3)^.

Así, se consideraron las fases de interpretación y descripción de los datos de este estudio, tales como: familiarizarse con los datos, generar códigos iniciales, buscar temas, revisar temas, definir y nombrar temas, así como producir el informe de análisis^(10)^. De esta manera, se realizó un análisis de las declaraciones a partir de las grabaciones y los datos se organizaron en categorías para poder tratar mejor el tema.

Cabe destacar que los participantes fueron identificados con la letra E de enfermero y recibieron una numeración secuencial en números arábigos, garantizando así el anonimato de las declaraciones. De este modo, se les denominó E1, E2 y así sucesivamente. No se utilizó inteligencia artificial.

### Aspectos Éticos

El estudio fue enviado y aprobado por el Comité de Ética en Investigación del Hospital seleccionado de la Universidad Federal del Triángulo Mineiro (HC-UFTM), según el número de dictamen 6.931.316 de 2024.

## RESULTADOS

Participaron en el estudio 13 enfermeros, con predominio del sexo femenino, 12 (92,3%), con edades comprendidas entre los 27 y los 45 años, con una media de 36,8 años.

En cuanto a la procedencia, la mayoría, siete (53,8%), era del estado de Minas Gerais, seguido de Bahía, dos (15,3%), São Paulo, dos (15,3%), Pernambuco, uno (7,7%), y Maranhão, uno (7,7%). El tiempo de actuación de los participantes en el sector de enfermería quirúrgica varió de seis meses a nueve años, con una media de 2,7 años.

El tiempo de formación fue de seis meses a 20 años, con una media de 11,7 años. En cuanto al trabajo en otros sectores del hospital, siete (53,8%) afirmaron no haber trabajado y seis (46,1%) afirmaron haber trabajado en otros sectores, como neurología, ortopedia, pediatría, urgencias para adultos y niños y UTI coronaria, para adultos y neonatal.

Cabe destacar que, de todos los enfermeros participantes, 11 (84.61%) tenían una especialización Lato sensu en diversas áreas, como Enfermería Quirúrgica, Urgencias y Emergencias, Cardiología, Terapia Intensiva y otras; además, cabe señalar que muchos profesionales tenían más de una especialización.

A partir de los datos de las entrevistas, se enumeraron categorías para organizar los resultados, a saber: “Factores asociados a la competencia en la gestión de conflictos”; “Causas de los conflictos” y “Estrategias para la gestión de conflictos: individuales y organizativas”.

### Factores Asociados a la Competencia en la Gestión de Conflictos

En esta categoría, cabe contextualizar que los enfermeros participantes mencionaron que se enfrentan a diario a una multiplicidad de demandas y vínculos interpersonales relacionados con el equipo técnico subordinado, los pacientes con sus acompañantes y todos los demás profesionales que componen el equipo multidisciplinario, ya que recae sobre su responsabilidad toda la atención sanitaria que se presta en el sector, por lo que se les encomienda la función de liderar y gestionar todas las situaciones.

De este modo, los enfermeros describieron la competencia de gestión de conflictos como la capacidad de mediar, manejar, lidiar, suavizar y resolver las situaciones adversas que surgen durante el turno. Otros factores asociados mencionados fueron la necesidad de construir una relación interpersonal e interprofesional con imparcialidad y comunicación asertiva, como se evidencia en los siguientes relatos:

*La gestión de conflictos, creo que es la forma en que se lidia en el trabajo con las disputas que van surgiendo, es tratar de suavizarlas, ya sea con los propios compañeros, con los técnicos de enfermería o con otros profesionales.* (E6)

*Se trata de saber lidiar con las situaciones cotidianas, generalmente entre el paciente, con los profesionales y entre los profesionales. Tenemos que ser flexibles, tener una buena relación tanto interpersonal como con el equipo, así como una buena relación con el paciente, el acompañante y todos los profesionales del hospital y, sobre todo, tener una comunicación clara y ser imparciales, no podemos tomar partido ni tener preferencias, sino saber analizar la situación.* (E7)

La capacidad de identificar y conceptualizar el conflicto, así como comprender que su gestión constituye una competencia esencial para el enfermero hospitalario, resulta imprescindible, ya que previene el deterioro de las relaciones personales y reduce la necesidad de recurrir a instancias superiores de la dirección del hospital.

*No hay forma de evitar que suceda, pero esta gestión, en mi opinión, implica conseguir al menos... ¿Cómo puedo decirlo? No dejar que empeore, no dejar que el conflicto aumente, llevarlo adelante, a largo plazo. Intentar resolverlo allí de la forma más puntual posible.* (E2)

*Tienes que gestionarlo para que no se convierta en algo más grande, para que no adquiera otras proporciones. Creo que la gestión del conflicto es eso, intentar resolverlo de la forma más, digamos, rápida, para no crear sobre un conflicto otros conflictos u otras situaciones. Creo que sería eso.* (E1)

### Causas de los Conflictos

Se mencionaron las causas más comunes relacionadas con la aparición de disensiones/conflictos en el sector de enfermería quirúrgica, siendo la principal la sobrecarga de trabajo, como consecuencia del absentismo y las bajas médicas. Sin embargo, el equipo nocturno menciona que existe un factor agravante que corrobora esta sobrecarga, ya que durante su turno no cuenta con la presencia de estudiantes, residentes, médicos suficientes e incluso la propia responsable técnica (RT) del sector.

Además, otras causas de conflicto descritas se relacionaron con la perspectiva de injusticia del equipo técnico en relación con las funciones delegadas y una relación interpersonal discordante. Además, los participantes también informaron que, cuando los profesionales del equipo tienen problemas personales o doble vínculo laboral, llegan al trabajo cansados y estresados, lo que a menudo resulta en la transferencia de esa tensión a sus colegas o pacientes. Así, estos profesionales pasan a actuar de manera irrespetuosa, grosera, explosiva y, a veces, ignoran las demandas de los acompañantes, lo que contribuye a aumentar los roces y desacuerdos.

Asimismo, los acompañantes poco colaborativos también fueron citados como potenciadores de conflictos. Y, por último, otra causa relacionada con el equipo multidisciplinario es la falta de atención interprofesional, ya que la atención fragmentada y poco cooperativa genera fallas en la comunicación, principalmente entre médicos y enfermeros, como se explica en los testimonios:

*Algo que implica mucho manejo de conflictos es la división, la división de enfermería. Es una de las cosas que más he notado, por la falta de profesionales. Entonces, muchas veces necesitamos, ¿no?, tener una organización que termina no siendo como sería en la legislación, esta división, porque no tenemos suficientes profesionales.* (E4)

*Creo que por la noche hay más conflictos que durante el día. Durante el día hay más gente para ayudar, hay más profesionales. Llega el médico y lo dice todo. Llega el estudiante y lo dice todo. Llega el residente y lo dice todo. Llega el psicólogo, llega... Entonces, hay tantos profesionales que al final no se sobrecarga tanto al equipo con las quejas. Ahora, por la noche, solo nosotros escuchamos. Cuanto más vamos allí, más se quejan. Entonces no hay forma de llamar al médico, al residente, para estas situaciones. Entonces el equipo se cansa más, se sobrecarga más.* (E3)

*A veces, el técnico que trabaja en varios lugares ya llega estresado, y además tiene un problema familiar y lo descarga en el paciente.* (E11)

*Hay siete técnicos de enfermería para 44, 45 pacientes, es muy poco. Es muy poco personal para tantos pacientes. Así que no hay manera. Aunque quisiera, no puedo poner un técnico en cada sala.* (E9)

*Como hay mucha sobrecarga, veo que cada uno solo hace lo suyo y ya está, no hay colaboración ni unión para discutir los casos juntos y eso potencialmente lleva a discusiones, porque a veces uno entra en la función del otro.* (E5)

Ante todas estas causas enumeradas, muchos enfermeros participantes se dieron cuenta de que una postura autoritaria e impositiva solo aumenta y hostiga aún más estos roces:

*No me gusta ser autoritaria, tener que ser impositiva y decir: “Así es y punto”. Porque las cosas solo empeoran, se convierten en una bola de nieve. Una cosa lleva a la otra, ¿no?* (E2)

### Estrategias Para el Manejo de Conflictos: Individuales y Organizacionales

Se observaron múltiples modalidades de manejo, como las individuales, por ejemplo, el diálogo respetuoso y armonioso, la reflexión y la concienciación sobre los errores cometidos, así como el seguimiento de si ha habido una progresión en relación con los comportamientos equivocados. Del mismo modo, se observó que conceder privacidad y mantener una actitud cordial, es decir, hablar en voz baja, mirando a los ojos, hace que el enfermero consiga alcanzar una posición de respeto y autoridad:

*Entonces, lo que siempre intento hacer con todos ellos es mirarlos a los ojos, llamarlos a mi despacho para que hablemos. Nunca, nunca he hecho eso y creo, detesto a quienes lo hacen, llamar la atención delante de otras personas, estar en un grupo reunido, citar el nombre de alguien que no está en el grupo, como ejemplo.* (E12)

*Intento hablar y mirar a ambos lados. Es que ahora mismo no estoy pensando en nada específico que haya sucedido, pero si hay un conflicto, escucharé a ambas partes e intentaré ser lo más justa posible, ¿no? Con cada uno de ellos. Intentar gestionarlo de la forma más justa posible.* (E13)

*Creo que escuchar es fundamental, porque muchas veces el hecho de que la persona se desahogue ya la calma, ¿no? Y el diálogo, no hay forma de tener una buena relación, de resolver conflictos sin diálogo.* (E7)

*Observación y diálogo. Y escuchar. La cuestión de la comunicación en sí. Lo mejor es la comunicación interpersonal. Y respetuosa y, claro, observar después de un tiempo si ha mejorado.* (E8)

Por lo tanto, otras estrategias, enumeradas por los participantes como esenciales para la gestión de conflictos, son: el uso de la empatía; reuniones de alineación con el equipo; el uso de la toma de decisiones compartida y participativa con el equipo técnico en la definición de escalas, distribución de funciones, incluyendo la deliberación terapéutica con el usuario:

*Hacer verdaderas reuniones en grupo, con RT, y así ellos expresan lo que sienten, lo que se necesita en el sector, lo que está pasando, lo que creen que se debe mejorar. Entonces, todo se relaciona con el diálogo. Y algo que me parece muy importante es que estemos involucrados en todo el trabajo que se realiza allí. Porque para entender de dónde viene ese conflicto, esa ansiedad, hay que entender lo que el miembro del equipo está pasando con eso.* (E10)

*Ah, sí, hay que tener mucha resiliencia, hay que tener mucha paciencia. Vaya, demasiado, porque lidiar con los seres humanos es difícil. ¿No? Entonces, hay que tener mucha paciencia, a veces tenemos que pensar y repensar antes de hablar, tener empatía, para no decir una palabra que a veces lastima al otro, a veces ni siquiera es por mal, pero allí, en el calor del momento, terminas diciendo lo que no debías.* (E13)

*La escucha activa es muy importante. Saber cómo piensa la persona, por qué piensa así. ¿Qué quiere? No dejar que simplemente te pase el problema para que tú lo resuelvas. Yo pregunto: ¿cómo quieres que lo resuelva? ¿Cómo lo resolverías tú si estuvieras en mi lugar? ¿Qué harías?* (E12)

Por último, una estrategia peculiar adoptada por una de las enfermeras fue el uso de la oración al comienzo del turno, en un intento por unir al equipo y promover el respeto, la armonía, el trabajo en equipo y reforzar el enfoque en la excelencia del cuidado al paciente.

*Otro recurso que utilizo siempre es la oración y lo he incorporado a todos los turnos, porque en el momento de la oración también hablamos de la importancia de la unión, de ayudar al compañero, de la empatía, de permanecer durante todo el turno cogidos de la mano, para no sobrecargar a nadie, así que es una estrategia para unirlos y acabar rompiendo un poco el ambiente tenso que a veces se crea como consecuencia de estos conflictos.* (E11)

En algunas entrevistas se mencionaron estrategias organizativas puestas a disposición por el hospital y la dirección del sector, como conferencias y cursos en línea/presenciales sobre comunicación no violenta, acoso moral y/o sexual, gestión de equipos, liderazgo, además de seminarios web y plataformas con diversos contenidos relacionados con el tema.

Además, el sector promueve la educación continua sobre el trabajo en equipo y una reunión general mensual integrada por la RT, la jefa de unidad y un consejo de gestión compuesto por un representante de cada categoría profesional, con la función de llevar a la agenda las principales demandas de su grupo:

*El año pasado se acordó un curso aquí en todo el hospital sobre comunicación no violenta. Justamente para que todos desarrollaran esta habilidad de resolver las cosas hablando, ¿no? Y no de forma que se malinterprete u ofenda al otro o se sienta ofendido. Y era un curso obligatorio. Siempre hay algún curso, alguna formación continua. También existe la plataforma de formación continua. Así, todo el mundo tiene acceso a los cursos a través del correo electrónico institucional. Hay varios cursos gratuitos que se pueden hacer. Hay sobre acoso moral, sobre acoso sexual.* (E2)

*También ha llamado al equipo de trabajo, que viene la psicóloga a hablar con nosotros un par de veces, tanto el enfermero del trabajo como la psicóloga, porque el equipo mismo tiene momentos en los que está muy nervioso debido a los pacientes que son muy pesados, y entonces todo el mundo presenta muchos certificados médicos.* (E7)

Sin embargo, se observó que ninguna de estas estrategias organizativas se centra en el tema específico de la gestión de conflictos, lo que deja un vacío en la formación profesional de estos enfermeros.

*Varios cursillos. Hasta ahora no he visto ninguno sobre conflictos. Bueno, si lo ha habido, aún no he tenido la oportunidad de hacerlo; solo sobre comunicación, etc.* (E1)

A pesar de lo anterior, es importante destacar que los participantes señalaron que la gestión de conflictos se ha desarrollado a lo largo de los años por dos vías principales. La primera consistió en el aprendizaje con otros enfermeros y/o gestores más experimentados, destacados como referentes en la materia. La segunda fue a través de cursos, actualizaciones, especializaciones/MBA, ya que las estructuras de trabajo van cambiando a lo largo de los años, lo que repercute directamente en las relaciones personales y en la gestión de conflictos:

*Creo que solo cuando lo vivimos y tenemos a alguien a nuestro lado que es bueno gestionando conflictos. Vas aprendiendo, ¿no? Vas aprendiendo con la conducta de esa persona. Yo aprendí con algunas personas. Principalmente jefes de la división de enfermería que tuve cuando era RT.* (E9)

*Estos cursos también ayudan a... No es que te den una receta lista... Te dicen: haz esto con la fórmula A más B y lo conseguirás. No creo que sea ese modelo, pero te dan estrategias, formas de lidiar con eso, ¿no? Cómo hablar, cómo dar feedback a un empleado. Entonces, esa es una forma de lidiar con ello.* (E6)

Por último, es importante destacar el papel de las instituciones formativas para desarrollar y enseñar eficazmente a sus futuros profesionales el tema de la gestión de conflictos en sus planes de estudios. Contradictoriamente, los participantes mencionaron que tuvieron un contacto mínimo con el tema durante la licenciatura, lo que contribuyó a dificultar el desempeño en la resolución de conflictos en el día a día de la práctica laboral.

## DISCUSIÓN

El conflicto se define como la oposición real o percibida de necesidades, valores y/o intereses entre dos o más personas, causada por factores individuales u organizacionales, que da lugar a estrés no deseado, tensión o sentimientos negativos entre las partes en disputa^(11)^.

De este modo, la competencia en la gestión de conflictos es un tema cada vez más relevante en contextos laborales, especialmente en profesiones caracterizadas por altas exigencias emocionales y una intensa interacción humana, como la enfermería, ya que están expuestos a conflictos a diario.

A partir de los resultados obtenidos, se constató que los enfermeros consideran que la competencia para la gestión de conflictos es esencial para la práctica laboral, ya que está vinculada a la capacidad de mediar en una situación que surge durante su turno, ya sea con el equipo técnico, multiprofesional o con los pacientes y acompañantes. Además, muchos enfatizaron que también les corresponde a ellos suavizar la situación para que no tenga que llegar a instancias superiores de la dirección del hospital o tome proporciones inesperadas. Por lo tanto, dominar las estrategias de gestión de conflictos se vuelve vital para los enfermeros^(12)^.

Así, como señalan los resultados de este estudio, la gestión de conflictos no debe orientarse por el intento de evitarlos a toda costa, sino por la capacidad de reconocerlos como un fenómeno inherente a las relaciones humanas y, sobre todo, como una oportunidad de crecimiento organizacional y mejora de las prácticas colaborativas. El conflicto, cuando se maneja bien, puede ser un catalizador de cambios positivos, promoviendo la innovación, la revisión de procesos y el fortalecimiento de las relaciones interpersonales^(13)^.

Esto concuerda con lo que señalan los investigadores, es decir, cuando los conflictos que involucran a enfermeros se manejan mal o se ignoran, como cuando son repetitivos o cuando las soluciones son insatisfactorias, pueden surgir adversidades como baja productividad, entornos de trabajo negativos, aumento del estrés y la ansiedad, atención precaria al paciente, aumento del absentismo y la rotación, deterioro del trabajo en equipo, insatisfacción en el trabajo y daños a la reputación de la institución^(14)^.

Por lo tanto, es de suma importancia que los enfermeros sepan manejar estas situaciones, a fin de evitar que el caos se instale en el entorno laboral, ya que ellos están en la posición de supervisores del equipo técnico y canal de comunicación para el equipo multidisciplinario, dado que el paciente permanece todo el tiempo de su hospitalización directamente bajo los cuidados de enfermería.

Según los entrevistados, las situaciones que más conflictos causan, en la enfermería quirúrgica en concreto, son la sobrecarga de trabajo, la distribución de funciones, los profesionales del equipo con problemas personales o más de un vínculo laboral, los acompañantes resistentes y la escasa comunicación interprofesional, especialmente la relacionada con el equipo médico.

De acuerdo a lo observado por los investigadores, las causas más comunes de conflicto son las diferencias individuales, que incluyen variaciones de personalidad, valores, percepciones y estilos de trabajo entre los miembros del equipo. Además, destacan las cuestiones relacionadas con el poder y la autoridad, que también pueden desencadenar conflictos dentro de una organización, incluyendo disputas sobre la toma de decisiones, la distribución de responsabilidades, la jerarquía organizativa y la comunicación ineficaz^(2)^, lo que corrobora parte de los resultados del presente estudio.

De este modo, en lugar de considerarse un obstáculo, el conflicto puede revelar divergencias legítimas de opinión, valores o prioridades entre los profesionales que, cuando se discuten de forma respetuosa y estructurada, contribuyen a la construcción de consensos más sólidos y decisiones más equilibradas. Por lo tanto, es fundamental crear espacios seguros para el diálogo, estimular la escucha activa y desarrollar habilidades comunicativas que favorezcan la empatía y el entendimiento mutuo.

Teniendo en cuenta las diversas causas expuestas, es necesario destacar las diferentes estrategias enumeradas para la gestión de estos conflictos. Entre ellas, se destacaron estrategias de carácter individual, como la promoción de un diálogo armonioso y respetuoso, la explicación del error acompañada del reconocimiento de los profesionales que lograron superarlo, el mantenimiento de una postura discreta y cordial en los momentos de conversación y evitar actitudes autoritarias e impositivas como medio de resolución.

En este sentido, queda claro que la resolución colaborativa está en consonancia con las evidencias ya publicadas, que indican que, en entornos de alta presión, como el sector de la salud, la cooperación y el diálogo abierto son cruciales para manejar eficazmente los conflictos^(15,16)^.

Así, los participantes evidenciaron la importancia de promover reuniones de alineación y adoptar una postura democrática en el momento de las deliberaciones y la elaboración de los turnos de trabajo, con el fin de que el equipo técnico se sienta parte de esa organización laboral y no tenga una percepción de injusticia o favoritismo en las decisiones y acciones tomadas por el enfermero gestor. Para ello, el uso de recursos de gestión participativa y democrática permite equilibrar los intereses de las partes en conflicto, ayudando a los gerentes de enfermería a lidiar con situaciones en las que no es posible lograr la plena satisfacción de todos los involucrados^(16,17)^.

Además, los enfermeros mencionaron como útiles algunas estrategias organizativas que contribuyeron a la gestión de conflictos, como conferencias, cursos, seminarios web, formación continua y reuniones con el consejo de gestión. Sin embargo, ninguna de estas iniciativas estaba dirigida específicamente al tema de los conflictos, lo que pone de relieve una laguna formativa en esta área.

Este hecho es preocupante, teniendo en cuenta la necesidad de implementar procesos educativos orientados a la resolución de conflictos. El dominio de las técnicas de negociación y oratoria permite un ambiente de trabajo más armonioso y productivo, así como una asistencia al paciente de mayor calidad^(18)^. Así, los hallazgos también corroboran las evidencias de un estudio realizado con estudiantes de medicina y enfermería, en el que se destacó la simulación en enfermerías como estrategia para el desarrollo de competencias esenciales, entre ellas la negociación de conflictos^(19)^, considerada vital para la práctica clínica.

Por lo tanto, se observa que es imprescindible el esfuerzo institucional en esta dirección, ya que las organizaciones de salud enfrentan una escasez continua de personal y una demanda creciente de pacientes. Así, dotar a los enfermeros de competencias para la gestión de conflictos puede contribuir a mitigar el estrés y reducir la rotación profesional^(15)^.

Además, cuando los conflictos se resuelven de manera constructiva, se establece un ambiente más estable y motivador, en el que los profesionales se sienten valorados y comprometidos con sus funciones^(20)^, lo que fortalece la capacidad del hospital para atraer y retener talentos calificados, esenciales para el funcionamiento eficaz de las unidades de salud.

La recurrencia de estos temas en diversos estudios sugiere que la capacidad de los enfermeros para gestionar los conflictos de manera eficaz puede depender no solo de factores organizativos, sino también de características individuales^(21,22)^.

Sin embargo, se sabe que estas características no son innatas, por lo que los profesionales deben desarrollarlas a lo largo de su carrera, ya sea mediante cursos y especializaciones o conviviendo con otros enfermeros/gestores más experimentados que, por su experiencia, tienden a percibir con mayor claridad y manejar con mayor eficacia las situaciones de conflicto^(23)^.

En este sentido, cabe destacar la importancia de que las instituciones formativas inviertan en contenidos que preparen al estudiante para lidiar con los conflictos, lo que no se verificó en el presente estudio, dado que los enfermeros tuvieron poco o ningún contacto con el tema durante la licenciatura.

Estos datos contribuyen a los resultados de otras investigaciones que han puesto de manifiesto que los planes de estudio siguen centrándose predominantemente en contenidos técnicocientíficos, relegando las llamadas competencias relacionales a un papel secundario^(23,24)^.

Además, el reducido número de profesores con formación complementaria en liderazgo, comunicación y negociación limita el enfoque de este tema a discusiones teóricas, sin la debida articulación con la práctica. La cultura institucional jerárquica, frecuentemente presente en universidades y servicios de salud, también dificulta el desarrollo de espacios de diálogo sobre conflictos reales, reforzando la invisibilidad del tema^(24)^.

A esto se suma la sobrecarga curricular y la falta de metodologías activas, que podrían hacer viables simulaciones, reflexiones y estrategias concretas de resolución. De este modo, la gestión de conflictos sigue siendo una laguna en la formación, lo que puede comprometer la actuación del futuro profesional en contextos de alta complejidad relacional y organizacional, como la enfermería quirúrgica y el entorno hospitalario en general.

El estudio presenta limitaciones que deben entenderse a la luz de la lógica de la investigación cualitativa, cuyo objetivo no es la generalización de los resultados, sino la comprensión profunda de un fenómeno en su contexto específico. La participación de 13 enfermeros resultó suficiente para alcanzar la significación de los datos, lo que permitió identificar patrones y singularidades en la percepción sobre la gestión de conflictos. Sin embargo, la realización de la investigación en una sola institución hospitalaria pública limita la diversidad de contextos organizacionales y culturales.

Otra limitación relevante es la ausencia de una caracterización detallada de la enfermería quirúrgica. A este respecto, cabe destacar la importancia de elaborar estudios que contengan información detallada sobre la descripción de la enfermería, con el número de camas, el perfil de los pacientes atendidos, la estructura física y los criterios de dimensionamiento del equipo de enfermería.

Aunque los participantes señalaron lagunas en la formación académica sobre el tema, el estudio no profundizó en el análisis de los planes de estudios de las licenciaturas en enfermería. Aunque estas limitaciones están presentes, no invalidan los resultados obtenidos, sino que indican aspectos que pueden explorarse en estudios posteriores, ampliando el alcance y la aplicabilidad de las reflexiones aquí desarrolladas.

La identificación de los factores asociados a la gestión de conflictos pone de manifiesto que esta competencia no se limita al ámbito técnico, sino que implica dimensiones éticas, emocionales y comunicativas, a menudo descuidadas en la formación y la práctica profesional.

Reconocer que el enfermero actúa como mediador en situaciones de tensión entre equipos, pacientes y familiares refuerza la necesidad de invertir en capacitaciones que desarrollen habilidades como la escucha activa, la empatía, la asertividad y la inteligencia emocional. Al sacar a la luz estas causas, el estudio contribuye a desnaturalizar los conflictos como meros efectos secundarios de la rutina hospitalaria, proponiendo un enfoque más crítico y propositivo que considera el contexto institucional y las dinámicas de poder involucradas.

Por lo tanto, el estudio contribuye a ampliar la comprensión de la gestión de conflictos como una competencia esencial para la práctica de la enfermería, con implicaciones directas en la calidad de la atención, la salud mental de los profesionales y la eficiencia de los servicios de salud. Al promover una reflexión crítica sobre los retos y posibilidades de esta competencia, el artículo refuerza la importancia del desarrollo de políticas institucionales y de una formación integral y una gestión institucional comprometida con el bienestar colectivo y la humanización de las relaciones en el entorno hospitalario.

## CONCLUSIÓN

El análisis de la percepción de los enfermeros hospitalarios sobre la gestión de conflictos como competencia profesional revela contribuciones significativas para el campo de la enfermería y la salud, especialmente en lo que se refiere a la valoración de las habilidades interpersonales y la construcción de entornos de trabajo más colaborativos y resilientes.

Las causas de los conflictos, a su vez, revelan la complejidad de las relaciones interpersonales en el entorno hospitalario, marcado por la sobrecarga de trabajo, las jerarquías rígidas, las fallas de comunicación y las divergencias de valores.

En este sentido, contar con una formación duradera sobre los temas de liderazgo y comunicación eficaz es fundamental para el perfeccionamiento de esta competencia y la sostenibilidad de los equipos. La eficacia de la gestión de conflictos en el entorno de enfermería depende de una combinación de habilidades individuales, experiencia práctica y apoyo organizacional. Para mejorar el clima laboral, la productividad y la calidad de la atención, es fundamental invertir en procesos educativos, promover una cultura de diálogo y cooperación, y desarrollar competencias de mediación desde la formación académica hasta la práctica profesional continua.

Las estrategias individuales, como el autocontrol y la búsqueda del diálogo, son fundamentales, pero insuficientes si no van acompañadas de acciones institucionales que promuevan una cultura organizacional basada en la transparencia, el respeto mutuo y la valoración de los profesionales. La implementación de políticas de gestión participativa, espacios de escucha y mediación de conflictos, así como programas de desarrollo profesional continuo, son ejemplos de medidas que pueden fortalecer la actuación de los enfermeros como agentes de cambio.

Además, es evidente la necesidad de desarrollar y profundizar en disciplinas durante la licenciatura que preparen a los futuros profesionales de manera asertiva para escenarios laborales complejos con grandes exigencias interpersonales.

## Data Availability

Todo el conjunto de datos que respalda los resultados de este estudio se ha publicado en el propio artículo.
